# Telling Students to Evaluate Does Not Make It Happen: Task Stage, Offloading Tendency, and Error Detection in AI-Assisted Student Writing

**DOI:** 10.3390/bs16091671

**Published:** 2026-09-17

**Authors:** Mengmeng Fan, Pengcheng Chang

**Affiliations:** School of Management, Zhengzhou University, Zhengzhou 450001, China; mmfan@zzu.edu.cn

**Keywords:** cognitive offloading, generative AI, academic writing, evaluative judgement, error detection, randomised experiment, higher education

## Abstract

Instructors can prescribe which part of a writing task a student may delegate to generative AI, but not how the student works. This four-arm randomised experiment separated them. At Site A, 744 undergraduates were randomised to one of four divisions of labour across four rounds of report writing—AI drafts and the student evaluates and revises; the student drafts and AI evaluates; the student drafts, AI revises, and the student adjudicates each change; no AI—with all AI withdrawn at Week 6 post-test, immediately after the final round (639 completed). A reduced three-arm replication at Site B randomised 126 students (109 completed). Both arrangements in which the student judged AI-produced text outperformed the arrangement in which AI judged student-produced text and the no-AI control (*d* = 0.35 to 0.36), whereas having AI evaluate was equivalent to no AI. The benefit was unevenly distributed (arm × tendency *F*(3, 629) = 7.25, *p* < 0.001), and logs showed students high in dependent tendency re-outsourcing their assigned evaluation (*b* = 0.072, *p* < 0.001). Satisfaction carried no information about error detection (*r* = 0.015), while self-rated quality was weakly associated with writing performance. Prescribing a division of labour governs the assignment, not the cognition.

## 1. Introduction

A supervisor who receives a well-structured, fluently argued, fully referenced student manuscript now faces a question the finished product cannot answer: what did the student actually practise? Generative AI has made the written artefact a poor proxy for the cognition behind it, and the instructional response has converged on a division of labour—rules specifying which part of the work a student may delegate and which part must remain their own (Bearman et al., 2024). Such rules are attractive because they are enforceable at the level of the assignment. Whether they are effective at the level of the learner is a separate question, and it is the question this study addresses.

Three strands of literature bear on this question, and they have rarely been integrated empirically.

The first concerns what is offloaded. Cognitive offloading—the transfer of cognitive demand to an external resource—long predates generative AI and carries no fixed valence (Risko & Gilbert, 2016; Sparrow et al., 2011); its consequences depend on how the external resource is enrolled in the task (Grinschgl et al., 2023; Silva et al., 2026; Skulmowski, 2023). The two faces can be separated within a single sample: W. Fan et al. (2026) found that the cognitive relief students perceive from classroom AI use and the cognitive offloading they perform through it mediate their attitudes in opposite directions, the former complementary and the latter competitive. Work on generative AI has increasingly resolved a single dimension of use into two: engagement in which the learner keeps the cognitive lead and uses the system as scaffolding, and engagement in which the learner transfers the judgement itself (Y. Fan et al., 2025; Zhu et al., 2026). The same move has been made independently on engagement outcomes: Y. Wang et al. (2026) separated reflective from thoughtless AI use and argued that research has attended to how much students use AI at the expense of how they use it. That distinction predicts downstream outcomes better than frequency of use does. It also produced a finding whose implication has not been tested: the two forms of engagement felt equally satisfying in the moment despite opposite downstream associations, which led to the conjecture—explicitly labelled by its authors as consistent with, but not demonstrating, detection failure—that learners cannot tell from experience which kind of use they are engaged in (Zhu et al., 2026).

The second strand concerns where in a task the offloading occurs. Lai et al. (2026) decomposed writing into drafting, evaluating, and revising and compared three human–AI divisions of labour in a quasi-experiment with 101 EFL undergraduates. Having AI draft while the student evaluated and revised outperformed the more familiar arrangement in which the student drafts and AI evaluates, and both outperformed writing without AI. The peer-review results were more pointed still: students who had been assigned the evaluating role for four rounds were no better at identifying problems than students who had used no AI at all. The stage that is delegated, on this evidence, matters more than the amount delegated. Three gaps remain: the design contained no condition in which AI performed the revision, which the authors identified as a limitation; no individual differences were measured; most consequentially for interpretation, evaluation by students in the evaluating role was prescribed by the assignment rather than established through observed behaviour. Process-tracing evidence has since made that last gap pressing. Synthesising 33 studies of learner interaction with AI-mediated feedback in second-language writing, Alghamdi and Alghizzi (2026) reported that higher-regulation learners engage in selective uptake and recursive evaluation of AI feedback, whereas lower-regulation learners more often accept it rapidly and with reduced evaluative engagement. Those profiles are observed, not assigned, which is exactly what leaves open whether prescribing the evaluative role does anything to them.

The third strand explains why the evaluating stage should be the one that matters. Evaluative judgement—the capacity to judge the quality of work, including one’s own and others’—is treated in higher education as a core capability rather than an ancillary skill (Tai et al., 2018), and feedback literacy research locates the learning value of feedback in the learner’s processing of it rather than in its receipt (Carless & Boud, 2018; Molloy et al., 2020). Both traditions have been revisited in light of generative AI, with the concern that a system able to deliver instant authoritative-seeming quality judgements removes the occasion on which a student’s own judgement would otherwise be exercised (Bearman et al., 2024). This literature supplies the mechanism but almost no experimental evidence. It does not tell us whether a student told to evaluate will evaluate.

Basic learning research suggests why evaluation in particular should resist delegation, while drafting and revising may not. Retrieval and the effortful generation of a response produce retention advantages over passive reception (Roediger & Karpicke, 2006; Slamecka & Graf, 1978), and conditions that slow acquisition while improving durable learning—desirable difficulties—are systematically undervalued by learners, who prefer the fluent option (E. L. Bjork & Bjork, 2011). Diagnosing what is wrong with a text is such a condition: it is slow, effortful, and unrewarding relative to receiving a diagnosis. Cognitive load accounts add a boundary, since removing extraneous load can help while removing the germane processing that constitutes the learning defeats the purpose (Kirschner et al., 2006; Sweller, 1988). In this case, the three writing stages are not interchangeable loads. Drafting and revising are largely productive operations that AI can shoulder without eliminating the learner’s cognitive work; evaluation is the stage at which the germane processing lives, so delegating it removes the learning rather than the burden. The prediction is therefore directional and theoretically motivated, not merely a contrast among conditions. It also implies the asymmetry we test in RQ3: if learners systematically prefer fluency and undervalue difficulty, the arrangement that teaches least may be the one that feels best, and self-report will not distinguish them.

Human–computer interaction research provides the closest methodological precedent. Cognitive forcing functions—interface constraints that require a person to commit to their own judgement before seeing the system’s recommendation—reduce over-reliance, at the cost of subjective experience: participants find them effortful and dislike them (Buçinca et al., 2021). This establishes that compelling engagement is possible, and it hints at the asymmetry central to the present study, since the intervention that improved performance was the one that felt worse. The paradigm, however, is one-shot decision-making with an unambiguous correct answer, not a multi-week learning task, and its outcome is reliance on advice rather than what the person retains.

### The Gap and the Present Study

Put side by side, the three strands leave a specific gap. What an instructor can prescribe is the *stage*; what the student brings is a *disposition* toward how AI is used. If the two interact, then a single rule produces systematically different outcomes for different students, and its average effect conceals the fact that the students the rule was written for may be the ones it fails. Supervisors observe exactly this: they require students to revise their own drafts, and find that some students develop while others forward the revision to the model.

We therefore randomised undergraduates to four divisions of labour across four rounds of authentic, graded report writing, withdrew AI entirely at post-test, and measured three objective outcomes rather than relying on self-report: unaided transfer performance, peer-review quality, and the rate at which students caught known errors planted in an AI-generated manuscript. Baseline dependent offloading tendency entered as a continuous moderator, and prompt-level logs supplied a behavioural indicator of whether the assigned evaluation was performed or re-delegated.

Three questions organised the design:

**RQ1 (stage).** Which stage of report writing, when delegated to AI, most affects independent writing once AI is withdrawn? We predicted that arms in which AI drafted (A) or revised (C) would outperform the arm in which AI evaluated (B), and that all AI arms would outperform the no-AI control (D) on transfer (H1a). On peer-review quality, we predicted that arm A would exceed B and C, and—following Lai et al. (2026)—that arm B would *not* exceed the no-AI control on problem identification (H1b).

**RQ2 (interaction; the study’s core).** Does the student’s dependent offloading tendency moderate the effect of the assigned stage? We predicted a reliable arm × tendency interaction such that the advantage of arm A weakens as tendency rises (H2), and that within arm A the proportion of prompts that re-delegate judgement rises with tendency, providing behavioural rather than self-report evidence for the mechanism.

**RQ3 (detection).** Can students detect what AI-generated text gets wrong? We predicted higher catch rates in arm A than in B and C (H3a); that catch rate would be uncorrelated or weakly correlated with immediate satisfaction and self-rated quality (H3b), which would test one behavioural implication of that conjecture—that the subjective experience of the work does not covary with how much of the text’s error the student actually caught; that dependent tendency would negatively predict catch rate after baseline ability was controlled (H3c). H3b concerns the informativeness of these two global judgements, not students’ metacognition about the detection task itself, which this design did not measure.

**Figure 1:**
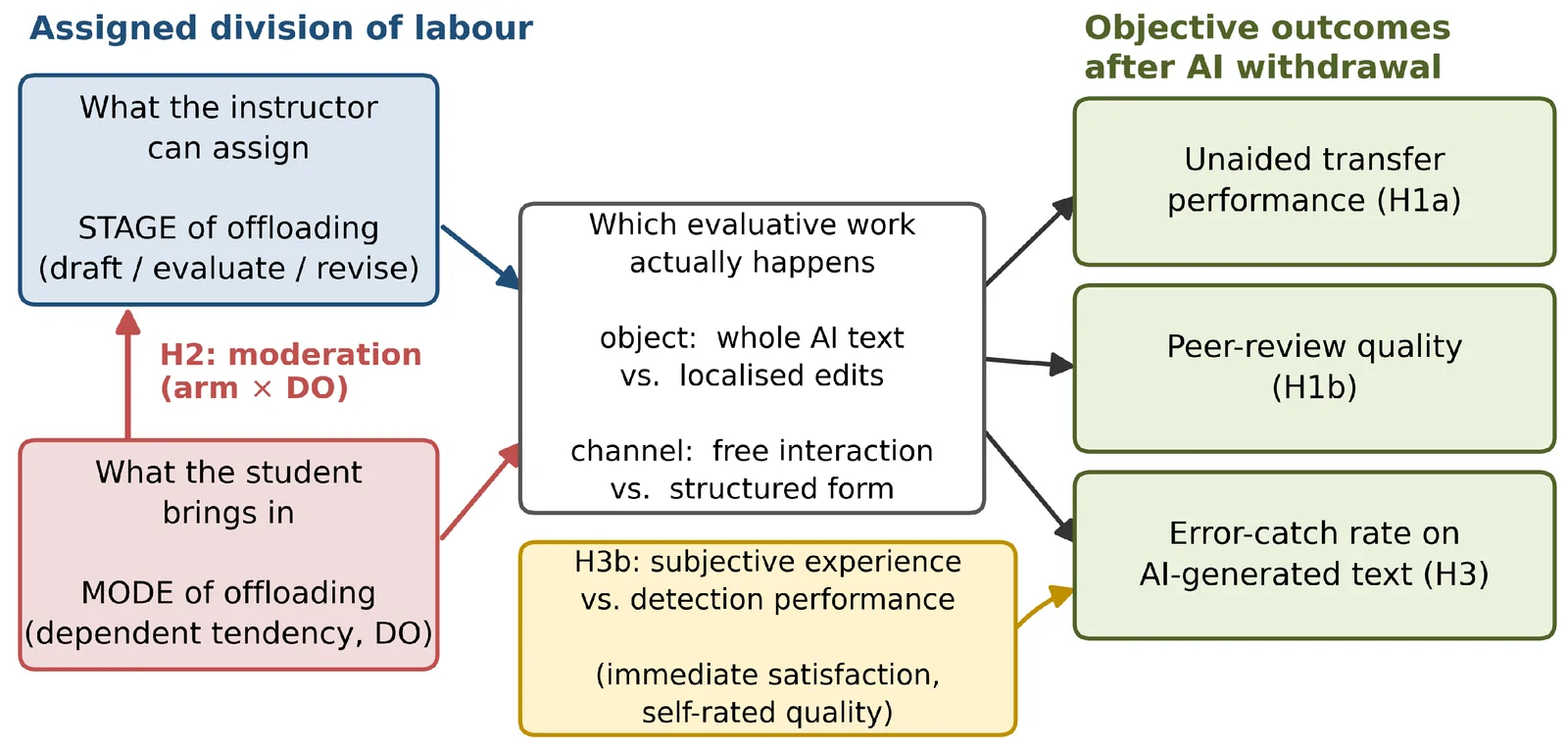
Conceptual model. The instructor prescribes the workflow; the student brings a disposition toward how AI is used. What the two jointly determine is not only who performs the evaluative work but what that work is directed at (a whole AI-generated text or a set of localised edits) and whether a channel remains through which it can be handed back to the model. Subjective experience is hypothesised to carry no information about what was missed.

Figure 1 sets out the resulting conceptual model. The two-dimensional framing of AI use is not new; several research groups have converged on it independently, and a scoping review of generative AI, cognitive offloading, and learner agency in higher education now maps the literature they have produced (G. Wang et al., 2026). What this study adds is a closed loop: correlational evidence replaced by randomisation, self-report by behaviour, and the question of which stage should be offloaded by the question of for whom the answer holds.

## 2. Materials and Methods

### 2.1. Design

The experiment was a four-arm, between-participants randomised design. The writing task was fixed as a three-stage cycle—draft, evaluate, revise—and the manipulation was which stage the AI performed:

- **Arm A**: AI produces the draft; the student evaluates it against the rubric and then revises it.
- **Arm B**: the student drafts; AI evaluates that draft against the rubric and returns feedback; the student revises accordingly.
- **Arm C**: the student drafts; AI returns a revised version directly, without presenting a separate evaluation; the student then adjudicates each change, accepting or rejecting it with a stated reason.
- **Arm D**: the student performs all three stages without AI.

Arms A, B, and D correspond to the three conditions of Lai et al. (2026), permitting direct comparison. Arm C supplies the condition those authors identified as missing, in which the revision itself is delegated. All three AI arms used the same model (Qwen 3.0, Alibaba Cloud), accessed at the provider’s default settings with web search disabled, and the same prompt-template pool; the only difference was what the student had to do with the AI’s output.

Two features of this operationalisation determine what the arms identify. First, the arms are workflows rather than an orthogonal decomposition of the three stages. In arm C, the model must appraise the student’s draft in order to revise it, even though that appraisal is never surfaced as feedback and the student never performs it; diagnosing one’s own draft is thus work the student does not do in arm C, as it is work they do not do in arm B. What separates arm C from arm B is the *direction* of the evaluative act: in arms A and C, the student renders judgement on text the AI produced, whereas in arm B, the AI renders judgement on text the student produced and the student executes it. Second, the *object* of that judgement differs between arms A and C. Arm A students appraised a whole AI-generated manuscript each round; arm C students appraised a set of localised changes. This difference yields a testable prediction: a task requiring whole-text diagnosis should build a capability that adjudicating discrete edits does not, and the error-detection outcome bears on it directly.

The task in each round was a report of 2500–3000 words containing a research question, a literature basis, an argument, and a conclusion—a miniature of a thesis chapter, and graded as part of the course. Theses themselves were not used. Randomly assigning some students to a less effective division of labour for work that determines whether they graduate places a degree at risk, and no ethics committee should approve it. The report preserved the authentic stakes and the same cognitive structure while removing that risk.

The platform constrained use to the assigned stage. Stages were released in sequence, so one could not begin before the previous had been submitted; the model was reachable only where the arm’s design placed it, the exception being arm A, where the student could consult the model freely once the draft had been returned; every request and response was logged. Within the platform, adherence was complete: every participant in an AI arm produced interactions, every participant in arm D produced none, and self-report agreed with the logs in every case.

Platform logs cover use within the platform. Compliance in arm D was verified against self-report as well as logs, with no case identified; consultation of a public model outside the platform would not appear in either record, as in any field experiment on a tool the participants can reach independently. The estimates are effects of the assigned arrangement under those conditions.

### 2.2. Participants and Sites

Participants were undergraduates at two institutions in a provincial capital in central China, referred to throughout as Site A and Site B; the institutions are not identified. Site A supplied the primary sample and ran the full four-arm protocol. Site B supplied a reduced three-arm conceptual replication (arms A, B, and D only, without the peer-review task).

The two sites differ in selectivity in a sense specific to the Chinese admissions system, in which undergraduate entry is determined almost entirely by a centrally administered examination and institutions admit within published score bands: Site B admits in the highest of those bands, Site A in a lower one. Both teach and assess in Chinese, and all tasks, rubrics, AI interactions, and instruments were in Chinese; the instruments were translated for reporting only.

Participants were third-year undergraduates majoring in e-commerce, a programme classified under different faculties at the two institutions; we used that classification as a stratum in the randomisation. The course ran across twelve class sections at Site A and four at Site B, taught by six instructors in total, and the study was conducted in the spring semester of the 2024–2025 academic year. The two sites were closely comparable on baseline experience with generative AI. The consent protocol collected the minimum personal information the analysis plan required, which did not extend to age or gender. Appendix B reports the composition figures.

Students were taught in intact classes, so allocation was nested within teaching units. Assignment was at the individual level within stratum rather than by class, which prevents class from being confounded with each arm but also means students in different arms sat in the same room; any resulting contamination would attenuate differences between arms rather than manufacture them. Class identifiers were not retained in the analysis dataset, so no model represents class- or instructor-level dependence, and faculty classification, which has two categories, cannot stand in for it. Because assignment was individual, a difference shared by all students in a class does not bias the contrasts between arms; residual correlation within classes or instructors could, however, make their standard errors too small.

**Figure 2:**
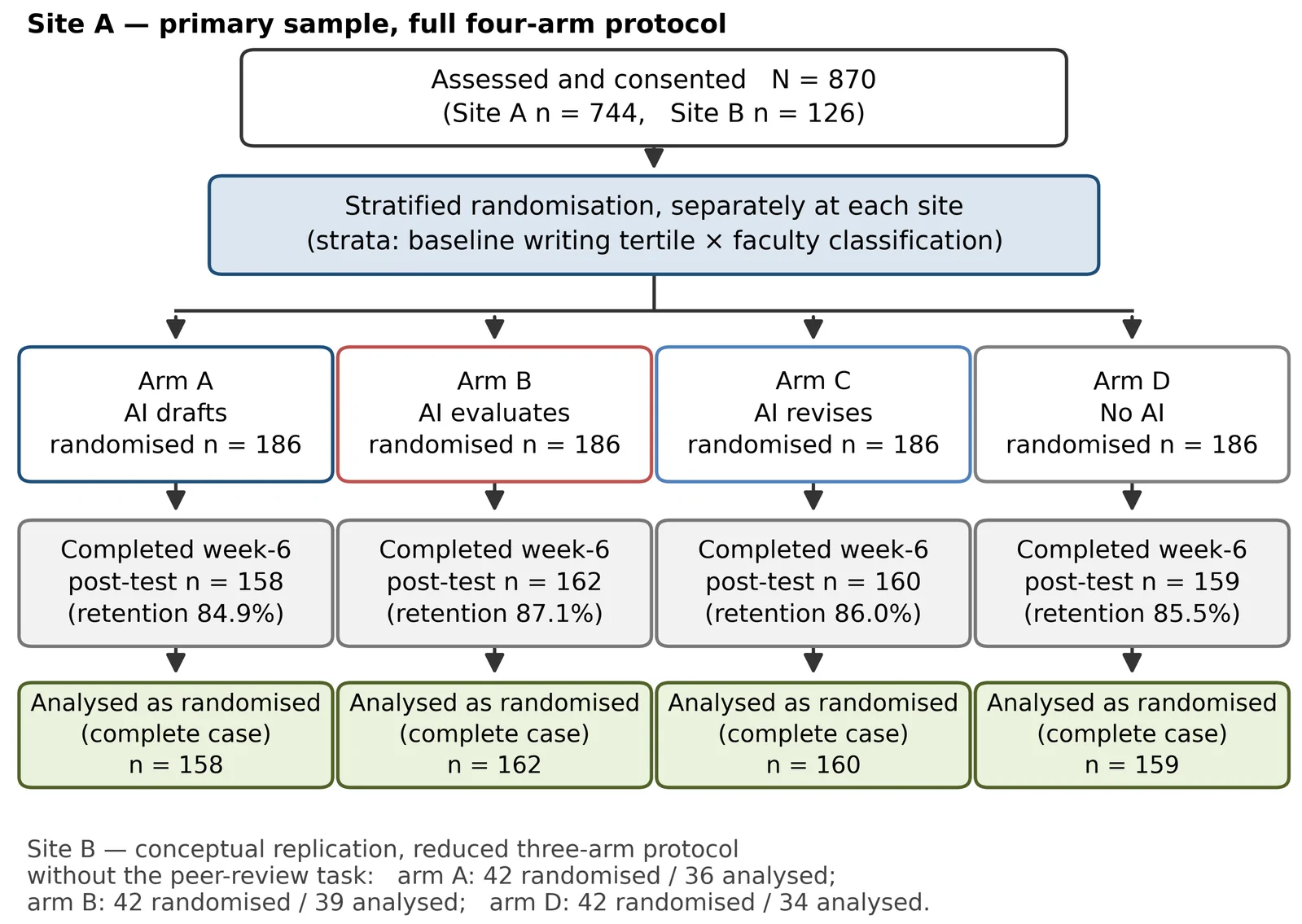
Participant flow. Randomisation was carried out separately at each site, within strata of baseline writing tertile and faculty classification; Site B ran a reduced three-arm protocol without the peer-review task. Participants are analysed in the arm to which they were randomised; because outcomes are measured only at the post-test, the primary analyses are complete-case analyses and the 105 Site A participants who did not complete it do not enter the outcome models.

At Site A, 744 students consented and were randomised, totalling 186 per arm. At Site B, 126 students consented and were randomised, totalling 42 per arm. Retention at the Week 6 post-test was 84.9% to 87.1% across arms at Site A (639 analysed) and 81.0% to 92.9% at Site B (109 analysed). Figure 2 reports the full participant flow.

The primary sample was placed at the less selective site for two reasons. The interaction test required roughly 520 completers, which the smaller cohort at Site B could not supply; locating the main conclusions in the population where AI dependence is most pronounced increases their practical relevance. Because institution type confounds intake, curriculum, and AI access, all primary tests were conducted within site, and the single pooled analysis reported below treats the site as a fixed-effect covariate. Site B is described as a conceptual, not a direct, replication.

### 2.3. Randomisation and Blinding

Randomisation was carried out separately at each site, within strata defined by baseline writing tertile and faculty classification. The allocation sequence was generated by the course platform rather than by any member of the research or teaching team; no teaching staff had access to it, and allocation was concealed until a participant had completed baseline measurement. The resulting arms were of equal size and did not differ on any baseline measure (Table 1). The allocation procedure and the stratum-by-arm counts are presented in Appendix B.

Participants could not be blinded to their own condition since the workflows visibly differ, but they were not told the direction of any hypothesis. Raters were blind to both condition and phase. All submissions were stripped of metadata, AI traces, and outline residue and shuffled before rating. Raters completed rubric training and a calibration set of 12–15 scripts, and rating proceeded only after ICC(2,*k*) ≥ 0.75 was reached on that set. Each script was rated by three raters, with a fourth independent rating whenever two raters differed by two points or more. Inter-rater reliability in the final sample ranged from ICC(2,*k*) = 0.787 to 0.846 across outcomes and sites, and rater main effects were not reliable for any outcome; per-outcome coefficients are presented in Appendix B.

### 2.4. Procedure

**Week 1.** Informed consent, baseline measurement, rubric training, and platform training. Baseline writing ability was assessed with an 800-word research note written in 45 min without AI and rated by assessors blinded to condition and phase; it served as both a stratifying variable and a covariate. Trait measures were administered in randomised order. All participants received identical rubric training and completed calibration items afterwards, so that arms began with comparable evaluative knowledge.

**Weeks 2–5.** Four rounds of report writing, one topic per round, counterbalanced across participants. Within each round, the sequence draft → evaluate → revise → submit was fixed; arms differed only in which stage the AI performed. The platform logged all interactions.

**Week 6.** Post-test, entirely without AI, comprising the transfer task, the peer-review task, the error-detection task, and post-measures. The post-test followed immediately after the final intervention round rather than after an interval, so it measures performance once AI is withdrawn rather than retention over time.

### 2.5. Measures

**Transfer performance (primary outcome).** An independent report on a new topic of equivalent difficulty, written without AI and rated on a six-dimension rubric by assessors blinded to condition and phase, scored 0–24.

**Peer-review quality.** Within 25 min, participants evaluated a common sample manuscript. Ratings covered problem identification, problem explanation, and constructive suggestion, each scored 0–5, using the same operationalisation as Lai et al. (2026) to permit comparison; a score of 0 records a dimension the reviewer did not address at all. This task was administered at Site A only.

**Error detection.** All participants received the same AI-generated report of approximately 1200 words containing 12 known planted errors, four of each type: fabricated citations (well-formed references to non-existent sources, and real sources whose conclusions were misattributed), logical fallacies (hasty generalisation, reversed causation, circular argument), and evidence–conclusion mismatches (data pointing opposite to the stated conclusion, inferential strength unsupported by sample size). Fabricated citations were included because they are the failure mode supervisors encounter most often in practice (Walters & Wilder, 2023). Each planted item is verifiable independently of any rater’s judgement: a fabricated citation either does or does not correspond to an existing source, a misattributed one either does or does not represent what its source concluded, and the logical and evidential faults were constructed to instantiate named fallacies rather than to be judged defective on impression. Two researchers confirmed each item against its type definition before piloting. Participants marked problems and stated why within a time limit. Scoring therefore required human judgement rather than string matching: a marked passage counted as a catch only when the accompanying justification identified the planted fault, so that marking the right passage for an unrelated reason did not score, and false positives were counted by the same procedure. Each response was scored by a single trained assistant who was blind to condition, so no inter-scorer agreement estimate is available for this outcome. Scorer latitude was limited instead by a fixed key that specified, for each of the twelve planted errors, what a justification had to identify for the item to count as caught, which reduced every scoring decision to a binary judgement about one item against a stated criterion. That key is provided with the instrument, so any scoring decision can be checked against it. The instrument itself was calibrated in a pilot of 8–10 participants per arm: the target band for each error type was a catch rate between 0.2 and 0.8, and any item whose type-level rate exceeded 0.9 or fell below 0.1 was rewritten before the main study. The catch rate was the number identified out of 12, computed separately by type. False positives—correct passages marked as errors—were recorded and entered the models as a covariate so that indiscriminate marking cannot inflate the catch rate. The item statistics in the final sample confirm that this calibration held. The twelve-item instrument was internally consistent, KR-20 = 0.619; the four-item type subsets are too short to support a confirmatory claim, so all confirmatory detection analyses use the twelve-item total and results by error type are exploratory (Appendix B).

**Interaction logs.** Prompts were stored with full context. The behavioural indicator for RQ2 was the re-outsourcing ratio: the proportion of a participant’s prompts that ask the model to make or substitute a judgement (*what is wrong with this paragraph*, *just fix it for me*) rather than to explain or justify. The unit of coding was the individual prompt rather than the session log, and the ratio is the proportion of a participant’s prompts classified as re-delegating. Two coders independently coded a randomly selected 20% of all prompts; agreement on that subset was Cohen’s κ = 0.83, above the threshold set in the protocol, after which disagreements were resolved by discussion and one coder classified the remaining 80% using the agreed scheme. The scheme, with its decision rules and the conventions that settled borderline cases, is presented in Appendix A. Coders saw the prompt text and arm—arm is inferable from the workflow and could not be masked—but had no access to participants’ offloading scores or to any outcome measure. The ratio is informative in arm A, where the student’s evaluative work was carried out in free interaction with the model and could therefore be handed back to it. It is structurally zero in arms B and C for different reasons: in arm B the student had no evaluative task to delegate, whereas in arm C the adjudication was completed inside a structured platform form—accept or reject each change, with a stated reason—which provided no channel to the model. It is undefined in arm D. This difference between arms A and C is a property of the workflow rather than of the students, and it bears directly on what a design can enforce.

**Self-report measures.** Dependent and autonomous offloading tendency were measured with the four-item subscales of the instrument developed by Zhu et al. (2026), metacognitive monitoring with the same source, along with AI use frequency and faculty classification. Both subscales were internally consistent at both sites, with α and McDonald’s ω between 0.79 and 0.83 (Appendix B). Metacognitive monitoring enters as a composite score, and the two post-measures are single items; internal consistency is not defined for either.

### 2.6. Analysis

Sample size was fixed in advance by Monte Carlo simulation of the primary test, the arm × tendency interaction, which consumes three degrees of freedom and has substantially lower power than the corresponding main effect. At α = 0.05 an interaction of Cohen’s *f* = 0.20 reaches power of 0.82 with 130 completers per arm, and we recruited to 720 against that target. Appendix B reports the simulation parameters and the power of the remaining tests.

The analysis plan—the three planned contrasts, the smallest effect size of interest, the correction families, and the decision to test equivalence where a null would carry weight—was fixed before data collection and is provided in full as Supplementary Material. The plan was fixed internally within the research team and carries no third-party timestamp; these analyses are therefore pre-specified rather than preregistered. Every analysis added after the data were seen is identified as such where it is reported. Participants are analysed in the arm to which they were randomised, with no reassignment by compliance or by what they actually did. Because the outcomes are measured only at the post-test, the primary analyses are complete-case analyses conducted according to randomised assignment rather than intention-to-treat in the strict sense: 744 students were randomised at Site A and 639 contributed outcome data, so the 105 who did not complete the post-test are absent from the outcome models. The attrition analysis is reported in full below. Effect sizes are reported as point estimates with 95% confidence intervals, and *p*-values are not used as the sole basis for any conclusion. A smallest effect size of interest of *d* = 0.30 was set in advance; effects below it are reported on both scales even when statistically reliable.

H1a was tested by ANCOVA of transfer on arm with baseline as a covariate, followed by three pre-specified contrasts under the Holm correction: A versus B, C versus B, and the mean of the AI arms versus D. H1b was tested by MANOVA across the three peer-review dimensions, with univariate follow-ups. H2 was tested as the arm × centred tendency interaction, with autonomous tendency also in the model because the two are not opposite poles of one dimension; tendency was never dichotomised. Simple slopes and a Johnson–Neyman region of significance followed a reliable interaction. H3 comprised the arm effect on catch rate, the association of catch rate with subjective experience, and the individual-difference model. Multiple-comparison correction was applied within families—Holm for the contrast and peer-review families, Benjamini–Hochberg for the detection family—and not applied to the single pre-specified interaction test, a decision fixed in advance and reported here rather than a post hoc choice.

Where a null result carries interpretive weight, equivalence was tested rather than inferred from non-significance, using two one-sided tests against bounds of ±0.30 *d* (or ±0.10 for correlations). Attrition was analysed before outcomes were examined. Analyses used Python 3.12 (statsmodels, SciPy); the bootstrap used 5000 resamples under a fixed seed. The package websites are https://www.statsmodels.org/ and https://scipy.org/ (both accessed on 15 September 2026). The analysis plan and the analysis scripts are provided as Supplementary Material so that every reported estimate can be traced both to the specification that called for it and to the code that produced it; the participant-level data are subject to the access conditions set out in the Data Availability Statement.

### 2.7. Ethics

The principal ethical risk in this design is the power asymmetry between instructors and their own students, and the protocol addressed it structurally. Instructors had no access to allocation lists and performed no rating; allocation and data handling were carried out by third-party research assistants. Research ratings and course grades were kept physically separate, produced by different people through different procedures and never shared. The consent form stated that participation, non-participation, and withdrawal at any time without providing a reason would have no bearing on any course evaluation or on the student–instructor relationship. Students who declined were offered equivalent conventional writing instruction.

The four intervention rounds were graded coursework, marked against a single rubric applied irrespective of arm—completeness of content, depth of analysis, structure and expression, quality of visual presentation, and clarity of the research question and its conclusions—and contributed 20% of the course mark. Students in the control arm worked without AI assistance throughout, so allocation could have affected their marks on this component; every participant received the full training materials for the most effective division of labour once the study ended. Course grades were held separately from the research dataset and were never linked to it. Research ratings played no part in any course mark, were produced by different people through a different procedure, and were never shared with the instructor. The study’s own outcomes are unaffected in any case: transfer, peer review and detection were all assessed at the Week 6 post-test, produced without AI by every arm under identical conditions, and carried no course credit.

## 3. Results

### 3.1. Randomisation, Attrition, and Compliance

Randomisation produced balanced arms at Site A on every baseline variable: writing ability, *F*(3, 740) = 0.04, *p* = 0.990; dependent tendency, *F* = 0.39, *p* = 0.758; autonomous tendency, *F* = 0.51, *p* = 0.674; metacognitive monitoring, *F* = 0.48, *p* = 0.699 (Table 1).

Attrition was 14.1% overall and unrelated to arm, $\chi^{2}$(3) = 0.39, *p* = 0.943. Completers and non-completers did not differ on baseline writing ability, *t* = −0.42, *p* = 0.679; dependent tendency, *t* = 0.12, *p* = 0.907; autonomous tendency, *t* = −0.17, *p* = 0.862; metacognitive monitoring, *t* = −1.61, *p* = 0.107. Compliance in arm D was complete on both records, logs and self-report, agreeing in every case, so the sensitivity analysis the plan specified for non-compliance did not arise.

Completion was unrelated to arm and every baseline measure, and re-estimating the primary models with inverse-probability weights or after multiple imputation moves no estimate by more than 0.08 transfer points (Table 2; Appendix B).

### 3.2. H1a: Which Stage Was Offloaded?

**Figure 3:**
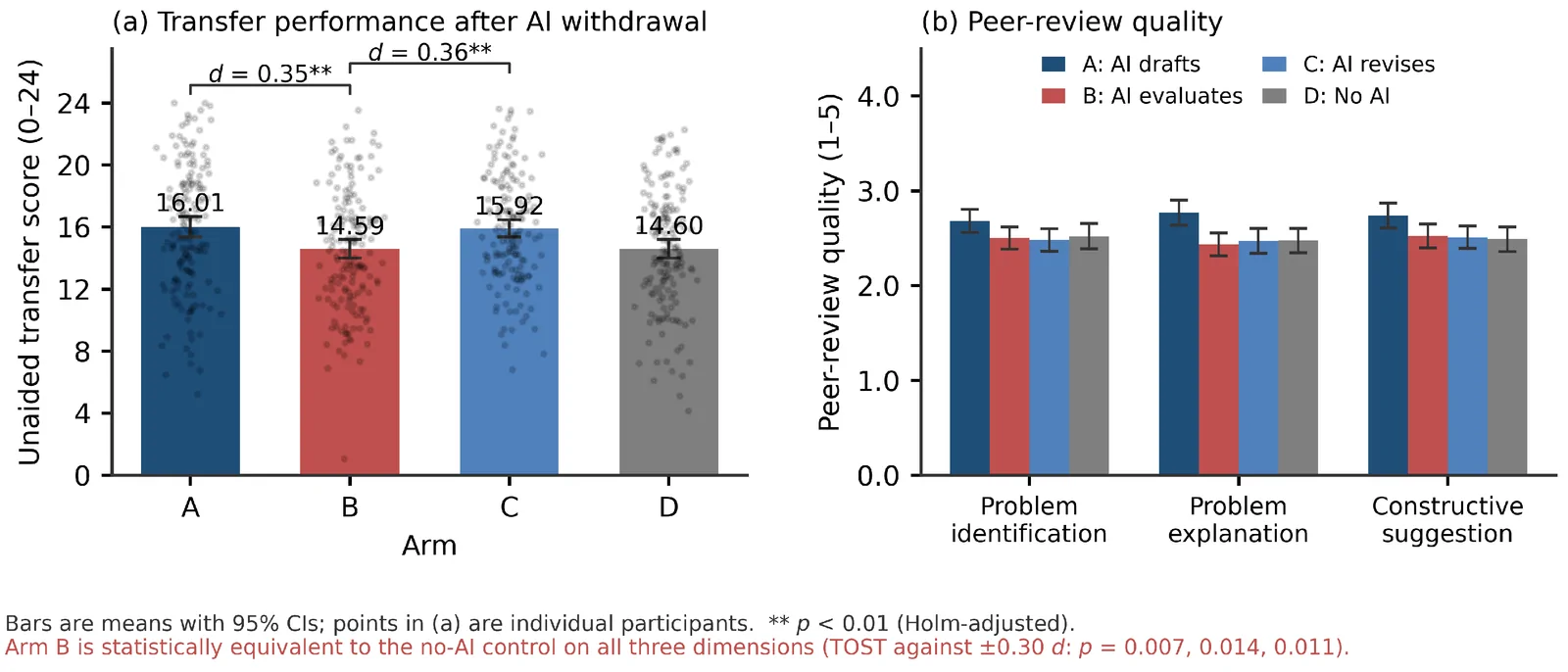
Stage effects at Site A. (**a**) Unaided transfer performance after AI withdrawal; points are individual participants, and bars are means with 95% confidence intervals. (**b**) Peer-review quality on three dimensions. Arm B, in which AI performed the evaluation, is statistically equivalent to the no-AI control on every dimension.

Transfer performance differed by arm, *F*(3, 634) = 8.18, *p* < 0.001, $\eta_{p}^{2}$ = 0.037. Means were 16.01 (*SD* = 4.18) in arm A, 14.59 (SD = 3.89) in arm B, 15.92 (SD = 3.56) in arm C, and 14.60 (SD = 3.86) in arm D (Figure 3a).

The three pre-specified contrasts (Table 3) were all statistically reliable. Having AI draft outperformed having AI evaluate, *d* = 0.35, 95% CI [0.13, 0.57], Holm-adjusted *p* = 0.004; likewise, having AI revise outperformed having AI evaluate, *d* = 0.36 [0.14, 0.58], *p* = 0.004; combining AI arms outperformed the no-AI control, *d* = 0.23 [0.05, 0.41], *p* = 0.012.

Those three contrasts do not by themselves test every component of H1a, which, as stated, requires each AI arm to exceed the control individually. The aggregate contrast of A, B, and C against D can be reliable while one of its constituents is not, and that is what happened here. All six pairwise contrasts appear in Table 3, each marked as planned or added, with the Holm correction applied across the six. Arm A exceeded the control, *d* = 0.35 [0.13, 0.57], adjusted *p* = 0.009, as did arm C, *d* = 0.36 [0.13, 0.58], adjusted *p* = 0.009. Arm B did not, *d* = −0.004 [−0.22, 0.22], adjusted *p* = 1.000. Arms A and C did not differ, *d* = 0.02 [−0.20, 0.24], adjusted *p* = 1.000.

**H1a is therefore supported in two of its three components and refuted in the third.** Both arrangements in which the student judged AI-produced text outperformed writing without AI at effect sizes above the smallest effect size of interest we set in advance. The arrangement in which AI judged student-produced text did not. The combined-AI advantage over the control, though reliable, is *d* = 0.23, below that threshold—this an average that, as the component contrasts show, is pulled down by including an arm that conferred no benefit at all.

That failure is the more informative half of the result, so we tested it as a claim rather than treating non-significance as evidence. Arm B was statistically equivalent to the no-AI control on transfer, *d* = −0.004 [−0.22, 0.22], TOST against ±0.30 *d*, *p* = 0.004, and arms A and C were statistically equivalent to each other (difference = 0.09 points, TOST *p* = 0.007). The equivalence test of B against D was specified in advance, on the ground that a null there would carry interpretive weight; the equivalence of A and C was not, and is reported as such. Four rounds of AI-generated feedback on the student’s own draft—the arrangement most often described in accounts of classroom practice (Alghamdi & Alghizzi, 2026; Bearman et al., 2024)—left students no better off at post-test than four rounds of writing with no assistance.

The benefit therefore does not attach to AI involvement as such. What distinguishes the two effective arms from arm B is the direction of the evaluative act: in arms A and C, the student rendered judgement on text the model had produced, whereas in arm B, the model rendered judgement on text the student had produced. Evaluation was not retained by the student in arm C in any absolute sense—appraisal of the student’s own draft was performed by the model there as in arm B—so the transfer data are consistent with the direction of judgement as the difference that matters, although the arms differ in other respects as well and do not isolate it. The detection data reported below show that this is not the whole story.

### 3.3. H1b: Peer-Review Quality

The multivariate effect of each arm across the three peer-review dimensions was reliable: Wilks’ Λ = 0.967, *F*(9, 1538.3) = 2.39, *p* = 0.011. Univariate follow-ups (Table 4) showed effects on problem explanation, *F*(3, 634) = 5.96, Holm-adjusted *p* = 0.002, $\eta_{p}^{2}$ = 0.027, and constructive suggestion, *F* = 3.58, *p* = 0.027, $\eta_{p}^{2}$ = 0.017, with problem identification not reaching the threshold, *F* = 2.37, *p* = 0.070.

H1b predicted that arm A would exceed both arm B and arm C. Both comparisons are reported across all three dimensions, with the Holm correction within the pre-specified family (Table 4). Arm A exceeded arm B on problem explanation, *d* = 0.41 [0.19, 0.63], adjusted *p* = 0.003, on problem identification, *d* = 0.24 [0.02, 0.46], adjusted *p* = 0.139; on constructive suggestion, *d* = 0.26 [0.04, 0.48], adjusted *p* = 0.125. Arm A likewise exceeded arm C on problem explanation, *d* = 0.35 [0.13, 0.57], adjusted *p* = 0.017, on identification, *d* = 0.26 [0.04, 0.48], adjusted *p* = 0.125; on suggestion, *d* = 0.28 [0.06, 0.50], adjusted *p* = 0.089.

**H1b is therefore partially supported.** All six contrasts run in the predicted direction, and the effect sizes are comparable in magnitude, but only the problem-explanation dimension survives correction against either comparison arm. Explaining what is wrong with a text is the dimension on which whole-text diagnosis paid off; identifying that something is wrong and proposing a fix did not separate the arms reliably with this sample size.

The finding that carries the theoretical weight is the comparison of arm B against the no-AI control. On problem identification, the two were indistinguishable: *d* = −0.03 [−0.24, 0.19]; the same held for explanation, *d* = −0.05, and suggestion, *d* = 0.04 (Figure 3b). Because non-significance alone would not support the claim, we tested equivalence directly: all three dimensions were statistically equivalent against bounds of ±0.30 *d* (*p* = 0.007, 0.014, and 0.011). This replicates the most counter-intuitive result reported by Lai et al. (2026) and strengthens it, since equivalence here is demonstrated rather than inferred from a failure to reject. Four rounds in which AI performed the evaluation left students no better at evaluating than four rounds with no AI at all.

### 3.4. H2: For Whom the Stage Effect Holds

The interaction of arm with dependent offloading tendency was reliable (*F*(3, 629) = 7.25, *p* < 0.001, $\eta_{p}^{2}$ = 0.033) in a model accounting for 24.5% of the variance in transfer. It survived the removal of all covariates (*F*(3, 631) = 6.77, *p* < 0.001) and the addition of faculty classification as a fixed covariate (*F*(3, 628) = 7.22, *p* < 0.001).

**Figure 4:**
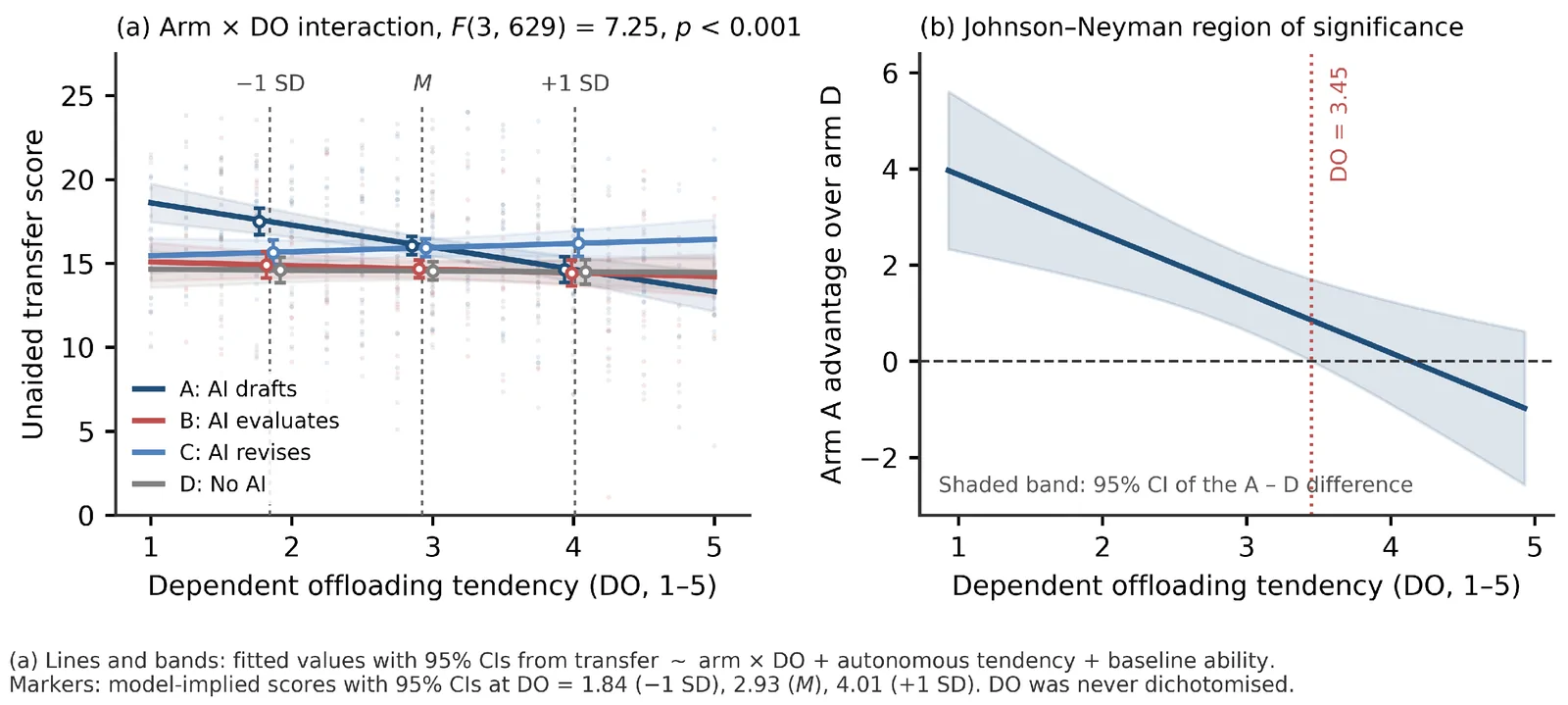
The stage effect depends on the student. (**a**) Fitted transfer performance against dependent offloading tendency by arm, with 95% confidence bands; the negative slope is confined to arm A. (**b**) Johnson–Neyman region of significance for the arm A advantage over the no-AI control, which ceases to be distinguishable from zero above a tendency score of 3.45.

Simple slopes locate the interaction entirely within arm A (Table 5). Among students who had an AI draft, a one-point difference in dependent tendency was associated with 1.41 fewer transfer points (95% CI [−1.97, −0.85], *p* < 0.001). In the other three arms, the slope was indistinguishable from zero: arm B, *b* = −0.07, *p* = 0.806; arm C, *b* = 0.23, *p* = 0.367; arm D, *b* = −0.10, *p* = 0.719 (Figure 4a). Tendency was measured rather than manipulated, so these slopes describe how the benefit was distributed across students, not what a change in tendency would cause. The slopes above are estimated within each arm, including the specification the analysis plan named. Deriving them instead as conditional effects of the interaction model provides closely similar values and identical conclusions—arm A, *b* = −1.32, 95% CI [−1.84, −0.81]; arm B, *b* = −0.22, *p* = 0.403; arm C, *b* = 0.25, *p* = 0.326; arm D, *b* = −0.05, *p* = 0.843—and it is that model’s fit that Figure 4a plots.

The Johnson–Neyman analysis converts this into the quantity an instructor would want (Figure 4b). At the low end of the tendency distribution, having AI draft was worth 3.97 points over writing without AI. The advantage declined monotonically and ceased to be distinguishable from zero above a tendency score of 3.45 on the five-point scale—a value 0.48 standard deviations above the sample mean of 2.93, and one exceeded by roughly a third of the sample. Above that threshold, the advantage of arm A was no longer distinguishable from zero at the precision this sample affords, which is not the same as its being zero; the point estimate remains positive, and the confidence interval includes values an instructor would care about. What the analysis supports is that the arrangement producing the largest average benefit produced no *detectable* benefit for the students who most habitually delegate judgement to AI.

The logs indicate what those students did with the assignment. Within arm A, where evaluation was the student’s responsibility, the proportion of prompts that re-delegated judgement rose with dependent tendency (*b* = 0.072 per scale point, 95% CI [0.054, 0.090], *p* < 0.001), and that proportion was itself negatively associated with transfer (*r* = −0.31, *p* < 0.001). Assignment to the evaluative role did not make evaluation happen; students high in dependent tendency forwarded it back to the model. Mean re-outsourcing in arm A was 0.28.

Re-outsourcing does not statistically mediate the tendency effect on transfer. The indirect path was not distinguishable from zero (*ab* = −0.03, bootstrap 95% CI [−0.40, 0.36]) and the *b* path was not reliable once tendency was in the model (*b* = −0.42, *p* = 0.863), while the direct effect of tendency within arm A remained (*b* = −1.40, *p* < 0.001). The prompt ratio therefore indexes the disposition rather than carrying its effect: it establishes that re-outsourcing occurs and scales with tendency, while the transfer shortfall runs through something other than this single measured behaviour.

### 3.5. H3: Detection and What Subjective Experience Tracked

**Figure 5:**
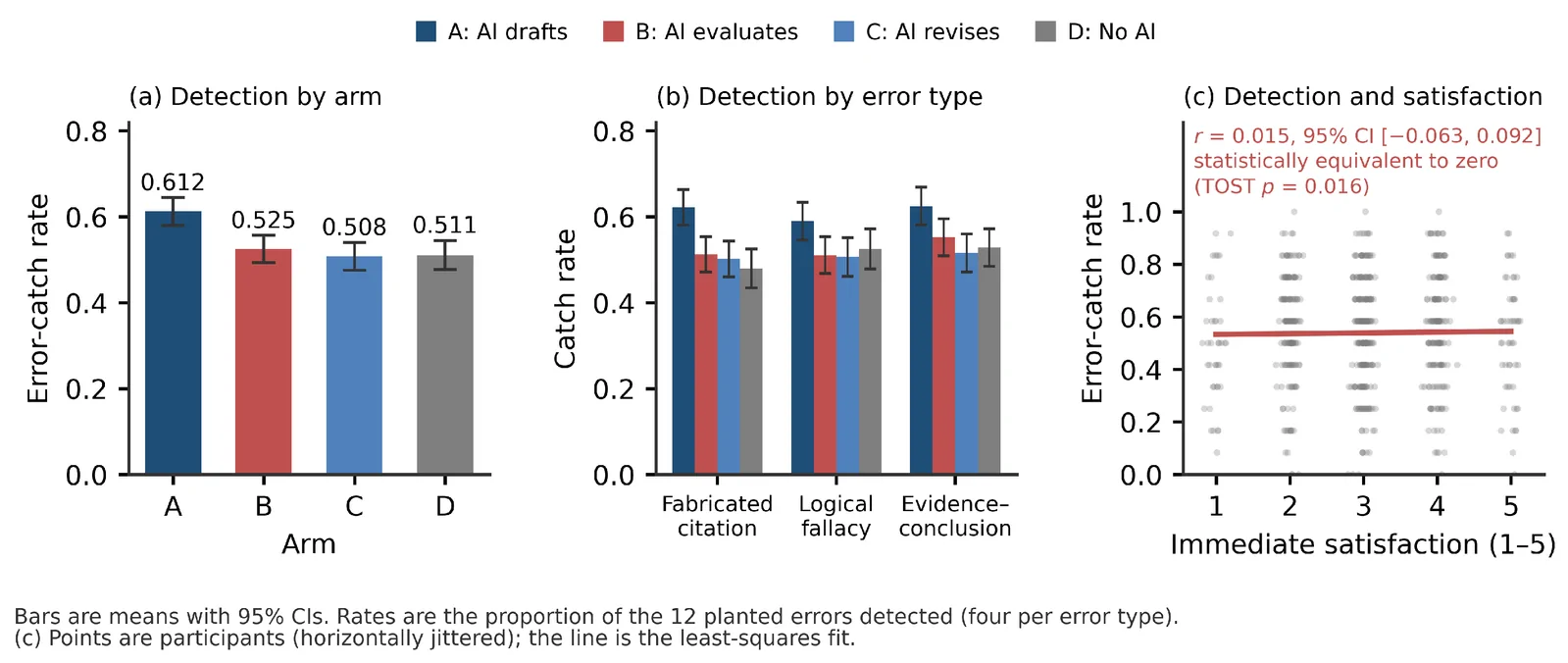
Detection of planted errors. (**a**) Catch rate by arm. (**b**) Catch rate within each error type. (**c**) Catch rate against immediate satisfaction with one’s own output; the association is statistically equivalent to zero.

Across 639 participants, the mean catch rate was 0.539 of the 12 planted errors. Arm affected detection (*F*(3, 633) = 10.64, *p* < 0.001, $\eta_{p}^{2}$ = 0.048), with false positives controlled. Arm A caught 0.612 of the errors, against 0.525 in arm B, 0.508 in arm C, and 0.511 in arm D (Figure 5a). All three comparisons of arm A against the others were reliable—versus B, *d* = 0.42 [0.20, 0.65]; versus C, *d* = 0.51 [0.28, 0.73]; versus D, *d* = 0.48 [0.26, 0.70]; all were Holm-adjusted (*p* ≤ 0.001) (Table 6). Broken down by error type—an exploratory division, since four binary items cannot support a confirmatory claim—the advantage held for each of the three and was numerically largest for fabricated citations (*d* = 0.51 [0.29, 0.74]), the failure mode with the most direct practical consequence (Walters & Wilder, 2023).

Two further models address the binary structure of the detection outcome. Analysing the catch rate as a count of successes in twelve trials rather than as a continuous score reproduces the arm effect, and entering the error type as a within-participant factor shows no interaction between error type and arm (Wald $\chi^{2}$(6) = 5.69, *p* = 0.459): the arm A advantage is common to fabricated citations, logical fallacies, and evidence–conclusion mismatches rather than driven by one of them (Appendix B).

This outcome separates arms A and C, whereas the transfer results did not. Arm C was statistically equivalent to the no-AI control on detection (*d* = −0.015, TOST *p* = 0.006) and equivalent to arm B as well (*d* = −0.085, TOST *p* = 0.027). Students who spent four rounds adjudicating the model’s edits ended the study able to write as well as students who had diagnosed whole AI-generated drafts and no better able than the control group to find what was wrong in one. The two arms differ in the way the design anticipated—arm A required whole-text diagnosis for each round; arm C made judgements about localised changes the model had already identified as worth making—though they differ in other respects as well, considered in the Discussion section. Delegating revision therefore carried no cost on transfer, but it did not build detection capability either, and any claim that revision can be safely offloaded has to be qualified by which outcome is being asked about.

The central test concerns whether either of the global judgements students made about their own work tracked how much error they had caught. Neither did. The catch rate was uncorrelated with immediate satisfaction with their own output (*r* = 0.015, 95% CI [−0.063, 0.092]), and equivalence against bounds of ±0.10 was supported (TOST *p* = 0.016). Its association with self-rated quality was marginal in the raw correlation (*r* = 0.080 [0.002, 0.156], *p* = 0.044) and disappeared once the arm and baseline ability were controlled (*b* = 0.005, *p* = 0.506) (Figure 5c). Likewise, immediate satisfaction carried no information after adjustment (*b* = 0.002, *p* = 0.789).

That these self-reports were uninformative about detection is not because they were uninformative in general: self-rated quality was weakly but reliably associated with transfer performance (*r* = 0.100, *p* = 0.012). The same judgements that carried some signal about how well students wrote carried none about how much of the planted error they had caught. Whether students could have reported their detection shortfall if asked directly is a separate question; answering it calls for confidence ratings on the detection items themselves, or an estimate of how many errors the student believed they had caught.

Individual differences ran in one direction only (Table 7). Dependent tendency predicted lower detection (*b* = −0.042 per scale point, 95% CI [−0.057, −0.027], *p* < 0.001). Neither autonomous tendency (*b* = −0.004, *p* = 0.610) nor metacognitive monitoring (*b* = 0.007, *p* = 0.571) predicted it. The students least likely to catch what AI got wrong were those most disposed to delegate judgement to it, and their self-assessed monitoring capacity provided no protection.

### 3.6. Robustness and Conceptual Replication

The interaction was not an artefact of model specification. It held without covariates and with faculty classification added as a fixed covariate, and there was no evidence that arms differed in the effect of baseline ability (*F*(3, 631) = 0.26, *p* = 0.854).

At Site B (*n* = 109 completers across arms A, B, and D), the stage main effect reproduced in direction and magnitude: arm A scored 16.60 (3.96), arm B 14.67 (3.13), and arm D 14.59 (4.27), providing *d* = 0.54 [0.08, 1.00] for A versus B and *d* = 0.49 [0.01, 0.96] for A versus D—estimates were as large as those at Site A, with the omnibus arm test at *F*(2, 101) = 2.85, *p* = 0.063. The interaction did not reach significance at Site B (*F*(2, 101) = 0.98, *p* = 0.378), and the within-arm slopes were correspondingly imprecise (arm A, *b* = −0.17, 95% CI [−1.74, 1.41]). With roughly 35 participants per arm, this test had little power to detect an interaction of the observed size; the Site B result is directionally consistent but inconclusive with respect to moderation.

A pooled analysis of the three common arms also supports moderation, with each site entered as a fixed effect rather than a random one, since two sites cannot identify a variance component. The arm × tendency interaction held (*F*(2, 579) = 7.00, *p* = 0.001), while the site itself and its interaction with the arm contributed nothing (Appendix B). The analysis was not pre-specified and is exploratory; two sites are not a sample of institutions, so the pooled estimate is not evidence for any wider population.

Finally, dependent and autonomous tendency correlated at *r* = −0.31, confirming that they are related but distinct rather than opposite poles of one dimension, which is why both entered the models.

## 4. Discussion

**Figure 6:**
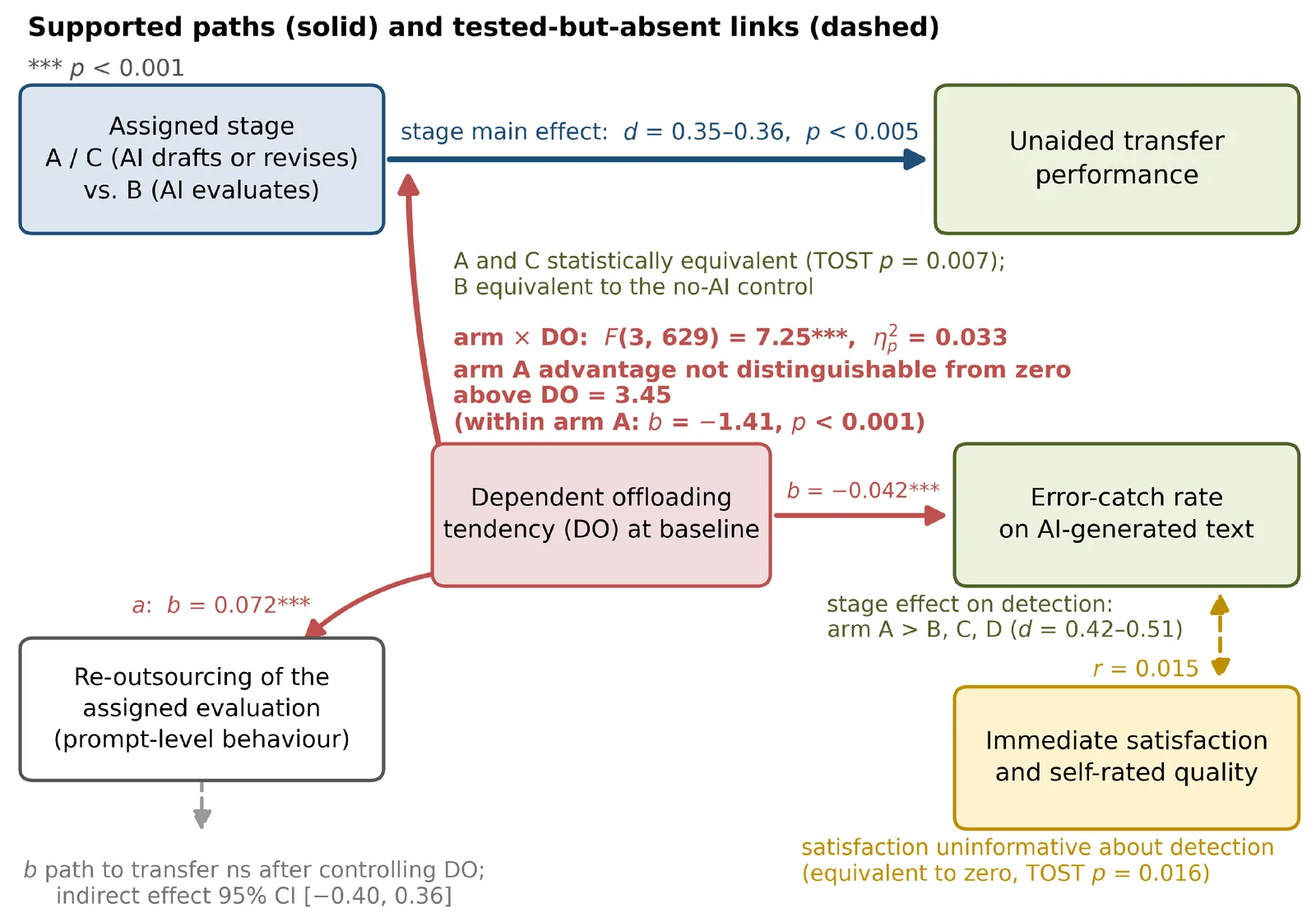
Results model. Solid paths were supported; dashed paths were tested and not supported. Re-outsourcing scales with dependent tendency but does not carry the effect on transfer, and error detection is unrelated to subjective experience.

Three findings, taken in order, describe a mechanism, as summarised in Figure 6.

**Which stage is delegated determines what the Week 6 outcomes look like, and the two outcomes do not agree.** On transfer, delegating drafting and delegating revision produced equivalent benefits, and both exceeded delegating evaluation, which was itself equivalent to using no AI. What separates the two effective arms from arm B is the direction of the evaluative act: the student judged the model’s text rather than executing the model’s judgement of theirs. This aligns the stage question with what evaluative-judgement research would predict (Bearman et al., 2024; Tai et al., 2018), with the qualification that literature has not previously been able to supply, since here the arrangement was randomised rather than observed.

Calling this *retaining evaluation* would overstate what the arms separate. Appraisal of the student’s own draft was performed by the model in arm C as much as in arm B; the difference lies in what the student had to do afterwards. The design does not orthogonally decompose the three stages, and the transfer result concerns the direction of judgement rather than the possession of a stage.

The detection results then show that even this is incomplete. Arm C matched arm A on transfer but was statistically equivalent to the no-AI control on error detection. This extends Lai et al. (2026) in the direction those authors identified—the missing fourth condition—but the answer it returns is conditional rather than clean. Revision can be delegated without cost to how well students subsequently write, and not without cost to whether they can tell that an AI-generated text is wrong.

What produced that difference is not identified by this design, and the candidates are several. Arms A and C differ in at least four respects, any of which could carry the detection effect. Arm A required appraisal of a whole manuscript rather than of a set of localised changes. Arm A therefore presented more diagnostic material per round. The proportion of the text that was AI-generated differed between the arms. And arm A alone gave the student the occasion to go back over material already passed. Arm C is not a control for arm A stripped of one ingredient; it is a different arrangement, and only arm A separated from the control on this outcome. The first is the explanation the design was built to test, and it coheres with the peer-review pattern, where the advantage is concentrated on explaining what is wrong. That is not identification: distinguishing these accounts requires arms that vary the scope of appraisal while holding the rest constant. That is the experiment this result most directly motivates. A single conclusion about whether a stage is safe to offload is in any case not available: the answer depends on which capability is being protected, and a design optimised for writing quality can leave detection untouched.

The practical inversion is nonetheless the sharpest result for teaching. The arrangement most often described in accounts of classroom and supervisory practice (Alghamdi & Alghizzi, 2026; Bearman et al., 2024)—the student drafts and the AI provides feedback—was the one arrangement that produced no measurable benefit on any of the three objective outcomes. It is not that this arrangement is mildly less effective. On these outcomes, it is indistinguishable from providing no AI at all.

**Assigning the evaluation does not make evaluation occur.** The interaction shows that the benefit of the best arrangement was concentrated among students low in dependent offloading tendency and was no longer statistically distinguishable from zero above a tendency score of 3.45, which roughly a third of the sample exceeded. The logs establish that the assignment did not control what it was meant to control: within arm A, the higher a student’s tendency, the more of their prompts asked the model to make the judgement they had been assigned. That the re-delegation occurred is established; that it is what produced the transfer shortfall is not, since the indirect effect was indistinguishable from zero. The point the logs support is that a rule can allocate a task without allocating the cognition, because the same tool that performs the delegated part remains available for the retained one.

This is where the stage literature and the mode literature meet, and the meeting point is not additive. The stage effect is not a property of the arrangement but of the arrangement crossed with the student. An average treatment effect of *d* = 0.35 conceals a range from a substantial benefit to none, and the students who receive none are precisely those whose habitual practice the intervention was designed to interrupt.

The mechanism goes only so far on this evidence. The prompt-level data establish that re-outsourcing occurred and scaled with tendency, which self-report could not establish. They do not establish that re-outsourcing is the channel: the indirect effect was indistinguishable from zero, and the transfer shortfall persisted with the ratio in the model. A single coded ratio is a coarse proxy for a disposition that plausibly also expresses itself in how long a student attends to feedback, how deeply they revise, and what they do with a judgement once made. The disposition matters and is visible in behaviour, and the pathway remains unidentified.

**Neither of the two global judgements students made about their work tracked how many errors they had caught.** Participants caught 0.539 of the planted errors on average and 0.612 in arm A, so they were far from unable to detect them. The catch rate was unrelated to immediate satisfaction—it was equivalent to zero, not merely non-significant—and unrelated to self-rated quality once arm and ability were controlled, even though self-rated quality was weakly associated with actual writing performance, *r* = 0.10. What the design establishes is therefore an asymmetry between two objects of judgement: the same students’ global self-assessments carried a small amount of information about the quality of their writing and none detectable about their detection performance.

What it does not establish is that students were unaware of their detection shortfall. Satisfaction with one’s output and a global quality rating are not metacognitive judgements about the detection task; a direct test would require confidence ratings on the detection items themselves, or an estimate of how many errors the student believed they had caught, and neither was collected. Students asked those questions might well have reported low confidence, and a dissociation between a global judgement and an item-level one is exactly what the metacognition literature would lead one to expect. The present result bears on the conjecture in Zhu et al. (2026)—that learners cannot tell from experience which kind of engagement they are in—by showing that one class of experiential signal, satisfaction with the product, does not covary with a performance dimension that matters. It does not settle whether a more targeted signal would. Supplying that test is the most direct extension of this study.

The asymmetry still carries a practical edge, because the judgements that failed to track detection are the ones students and instructors actually have to hand, and the error type where the gap between arms was largest—fabricated citations—is the one most likely to survive into a submitted manuscript (Walters & Wilder, 2023). It also connects to a broader pattern in how people relate to AI-assisted work, where the subjective experience of assistance and its actual consequences can move independently (Zhang et al., 2026).

Why the dissociation should take this particular form is answerable from what is known about how such judgements are formed. Metacognitive judgements are inferential rather than direct readouts of a cognitive state; they rest on cues whose validity varies with what is being judged (Koriat, 1997). A judgement is well calibrated when the cues it happens to recruit predict the criterion and poorly calibrated when they do not. Fluency is the cue most available to a student contemplating a finished text, and fluency is exactly what a competent language model supplies, irrespective of whether the content is sound. That fluency at encoding breeds confidence unwarranted by later performance is among the most robust findings in the study of self-regulated learning (R. A. Bjork et al., 2013; Koriat & Bjork, 2005), and the same logic predicts the pattern observed here: the cues that make a text feel satisfying overlap to some degree with those that make writing good, consistent with the weak association between self-rated quality and transfer, and are unrelated to whether a cited source exists, consistent with the absence of any association between either judgement and detection. That metacognitive accuracy is specific to the object being judged rather than a general trait is itself well established (Kelemen et al., 2000), and the null for trait monitoring reported above is what that literature would lead one to expect. On this reading, the dissociation is not an anomaly of AI-assisted work but an instance of a known regularity, met in a setting where its consequences are unusually costly (Messeri & Crockett, 2024).

A second question is why four rounds of evaluative work left detection unimproved in three of the four arms. The relevant account here is automation-induced complacency: when a system reliably supplies output of apparent quality, attention to that output declines, and the errors that escape are disproportionately those the system did not itself flag—errors of omission (Parasuraman & Manzey, 2010; Skitka et al., 1999). Arms B and C map onto this directly. In arm B, the student received a diagnosis and never searched for one; in arm C, the student ruled on changes the model had already located, so whatever it passed over was never brought into view. Neither arrangement gives an omission error the occasion to be found, which is what detection at post-test demanded. The implication is that the capability will not accrue as a by-product of use, and there is converging reason to expect this: people’s intuitive heuristics for judging AI-generated language are systematically wrong (Jakesch et al., 2023), and students working with a model on authentic tasks calibrate their confidence to its output rather than to its correctness (Kaplar et al., 2026). Building detection plausibly requires explicit instruction in critical AI literacy (Ng et al., 2021)—something no arm of this study provided, and something these results argue is not substitutable by experience in the evaluator’s role.

Three features of this result deserve emphasis. It is not a general miscalibration effect in the sense of Kruger and Dunning (1999), since the same participants’ judgements were partially calibrated about their writing. Trait metacognitive monitoring, measured at baseline, predicted neither detection nor the size of the dissociation. Detection was lowest among students high in dependent tendency, so the students who caught the least were also those whose habitual practice the arrangements were meant to interrupt.

### 4.1. Theoretical Implications

Compared with the learning literature, the pattern is more specific than a general claim that AI harms or helps. On transfer, delegating drafting and delegating revising were equally harmless, which is what a cognitive load account predicts if those stages are largely productive operations whose removal does not eliminate the germane processing (Sweller, 1988). Delegating evaluation was not harmless on any outcome, and the sharpest expression of this is that four rounds of AI feedback left students no better at diagnosing a text than four rounds without AI. Evaluation behaves as the desirable difficulty of the writing cycle (E. L. Bjork & Bjork, 2011): the stage whose effortfulness is the point, and whose removal is experienced as relief while the learning it carried disappears with it.

The detection contrast between arms A and C refines this, with the caveat entered above that the two arms differ in more than one respect. Both required the student to judge model-produced text, and both yielded the transfer benefit, but only arm A improved detection. The desirable difficulty plausibly lies in the unaided search for what is wrong rather than in the act of judging as such: adjudicating changes the model has already located is judgement without search, and it did not transfer to finding errors nobody had flagged. This is a hypothesis the present contrast is consistent with rather than a mechanism it isolates. What the contrast does establish is that the granularity at which a stage is defined is a substantive modelling choice: two arrangements that both count as “the student evaluates” behaved differently on one of the two outcomes, and a design treating evaluation as a single stage would not have shown it.

This locates the harm of generative AI in learning at a level below the tool and below the amount of use. What matters is which cognitive operation is displaced, and that operation is not visible in the artefact—which is precisely the supervisor’s problem. It also refines the two-dimensional framings that several groups have converged on (Y. Fan et al., 2025; Zhu et al., 2026). Those frameworks treat the mode of engagement as a property of the person. The present results show it is a person-by-situation product: the same disposition was inert in three of four arms and decisive in the fourth. A disposition to offload can only express itself where the design leaves a channel through which to offload. Work on automation trust shows a structurally similar pattern, with individual differences moderating susceptibility to complacency rather than determining it outright (Tang et al., 2026); that research concerns driving rather than learning, so the parallel is one of form rather than of content.

The comparison of arms A and C makes this concrete, and it is an observation the study did not set out to produce. In arm A, the student’s evaluative work took place in free interaction with the model, so re-delegation was available—and the students disposed to re-delegate did so, and lost the benefit. In arm C, the equivalent work was completed inside a structured form with no channel to the model, and the slope of tendency on transfer in that arm was flat (*b* = 0.23, *p* = 0.367). The disposition did not stop existing in arm C; it had nowhere to go. The channel in arm A was also a continuing one: a student’s working relationship with the model carried across the four rounds rather than restarting at each assignment, so a student who had established a habit of asking for verdicts did not have to establish it again. That is how students ordinarily work with such a system over a term, and it is part of what makes an assigned evaluation forwardable in practice rather than only in principle. The two arms differ in more than the presence of a channel and were not designed as a manipulation of it, so this is a lead for direct testing rather than a result. It is nonetheless the pattern to expect if enforcement rather than instruction is what makes the difference.

Finally, the detection result bears on the assumption of learner self-regulation that underwrites much of this field. Self-regulated learning models presume access to a monitoring signal (Zimmerman, 2002), and interventions from reflective prompting to self-assessment inherit that presumption. Here one such signal was uninformative in a specific and testable sense: satisfaction was statistically equivalent to uncorrelated with detection, while self-rated quality remained weakly informative about writing quality. Whether learners retain access to some other, more targeted signal about detection is precisely what this design leaves open, and it is the question a follow-up should ask. What the present data do show is that the signal most readily available—how satisfied a student is with the text in front of them—does not carry that information. Theories of self-regulated learning developed before generative AI may therefore need to specify which objects of monitoring remain accessible when the work is co-produced with a system whose errors are fluent, rather than treating monitoring as a single general capacity.

### 4.2. Practical Implications

The design implication follows from the third finding, with its scope stated carefully. Self-regulated correction, reflective prompts, and honour-code-style undertakings all assume the learner can infer from the experience of the work that something went wrong. These data show that one obvious basis for that inference—satisfaction with the product—carries no information about how much error the text still contains. They do not show that no such basis exists. The prudent design posture is therefore not to rely on the student’s global sense of the work having gone well, which is the signal these arrangements most often invoke and the one measured here. This is not to treat monitoring accuracy as fixed. Rewarding accurate monitoring rather than performance alone has been shown to raise both together (Liu et al., 2025), which indicates that calibration is something a design can aim at directly. Aiming at it, however, is a different intervention from assuming it, and it is the assumption that the present results undercut.

What follows is a preference for structure over exhortation. If the evaluative work is what has to happen, it has to be enforced by the environment rather than requested of the student—which is the logic of cognitive forcing functions (Buçinca et al., 2021), transplanted from single decisions into a multi-week learning task. Arm C is an instance of such a structure that happened to be in the design: the adjudication had to be entered as a form, there was no channel to the model, and the disposition that cost students the benefit in arm A was inert there. Requiring a committed judgement to be recorded before the model can be consulted, capping consultations during the evaluative stage, or making the evaluative artefact itself the assessed object are all mechanisms of the same kind, and none of them depends on the student noticing anything. The arm C result also carries its own warning, since that arrangement protected transfer without building detection: enforcement has to be aimed at the capability one actually wants. The cost that Buçinca et al. (2021) documented—such constraints feel worse—appears here in a sharper form: because neither global self-judgement carried information about detection, student satisfaction is not a usable signal for whether such a design is working, and optimising for it would favour arrangements that feel good over arrangements that produced the outcomes measured here. Student preference for AI involvement in scoring and feedback is itself under active study and varies with context and stakes (Yildirim-Erbasli et al., 2026); a design selected on that basis would be tracking something these data indicate carries no information about what students failed to detect.

For supervisors specifically, the results argue against the intuitive rule, with two qualifications the data insist on. Telling a student to write the draft themselves and let AI check it is the arrangement that failed on every outcome. Letting AI draft while the student diagnoses it produced gains on all three. Letting AI revise while the student adjudicates the changes produced the writing gain but left detection where it started, so it is the weaker of the two options if the concern is students accepting fabricated material.

The second qualification concerns whom each arrangement serves, and the two effective arms differ here in a way the average effects conceal. The advantage of arm A was concentrated among students low in dependent tendency and was no longer statistically distinguishable from zero above a tendency score of 3.45; in arm C, the slope of tendency on transfer was flat (*b* = 0.23, *p* = 0.367), so what benefit that arrangement conferred did not depend on the disposition. In other words, only arm A delivered its benefit selectively. The practical reading is therefore a qualified pair of recommendations rather than a single rule: arm A is the stronger arrangement on average and the only one that built detection, but it is the one whose gain the students it was written for are least likely to receive; arm C was insensitive to the disposition and left detection untouched. Either way, the disposition is worth measuring at intake rather than assumed, since it is what separates the students for whom the better arrangement works from those for whom it does not.

### 4.3. Limitations

Three limitations bound these claims. The arms are workflows rather than an orthogonal decomposition of the three stages, so the contrasts bear on the direction and object of the student’s evaluative act rather than isolating the offloading of any one stage, and the four respects in which arms A and C differ are not separated either. The inferential base is also narrower than the sample size suggests: the moderation rests on one site, since Site B reproduced the stage main effect at comparable magnitude but had too few participants per arm to test the interaction, and because class identifiers were not retained, residual correlation within classes or instructors is not modelled and may make the reported standard errors too small. And what can be said about mechanism is bounded in two ways. For example, the pathway from tendency to shortfall is unidentified, since re-outsourcing does not mediate it and the cognitive-agency and motivational constructs theorised to carry it (Zhu et al., 2026) lie outside the measures taken here. The detection findings concern the informativeness of two global self-judgements rather than students’ metacognition about the detection task, which would require item-level confidence ratings or estimates of errors missed (Fleming & Lau, 2014; Schraw, 2009).

## 5. Conclusions

Which stage a student offloads to AI does matter, and what has to survive the arrangement is the student’s own evaluative work on the model’s output—though the two outcomes measured here disagree about how much of it is enough, since adjudicating the model’s edits was sufficient for writing and insufficient for detecting error. An instructor who prescribes a division of labour has in any case prescribed an assignment, not a cognitive act, and the students most likely to convert the assignment back into a delegation are the ones the prescription was written for. Nor will their satisfaction with the result serve as a signal, since it carried no information about how much error they had caught. Designing for AI-assisted learning therefore means building environments in which the evaluative work cannot be forwarded, rather than instructions asking that it not be.

## Figures and Tables

**Table 1:** Baseline balance across arms at Site A (all randomised participants, *n* = 744).

	Arm A	Arm B	Arm C	Arm D	*F*(3, 740)	*p*
Baseline writing (0–24)	15.10 (3.15)	15.01 (3.29)	15.12 (3.01)	15.08 (3.16)	0.04	0.990
Dependent tendency (1–5)	2.97 (1.06)	2.92 (1.05)	2.86 (1.09)	2.96 (1.14)	0.39	0.758
Autonomous tendency (1–5)	3.06 (1.10)	2.93 (1.11)	3.03 (1.07)	3.05 (1.11)	0.51	0.674
Metacognitive monitoring (1–5)	3.04 (0.74)	2.99 (0.81)	2.96 (0.73)	3.04 (0.78)	0.48	0.699
Randomised	186	186	186	186		
Completed post-test	158	162	160	159		
Retention	84.9%	87.1%	86.0%	85.5%	$\chi^{2}$ = 0.39	0.943

Cell entries are *M* (*SD*).

**Table 2:** Sensitivity of the primary estimates to the treatment of missing post-test data (Site A, transfer performance).

Estimate	Complete Case (*n* = 639)	IPW (*n* = 639)	Multiple Imputation (*n* = 744)
A vs. B, mean difference	1.425	1.432	1.448 [0.585, 2.310]
C vs. B, mean difference	1.334	1.332	1.326 [0.521, 2.131]
A vs. D, mean difference	1.409	1.422	1.485 [0.606, 2.363]
C vs. D, mean difference	1.318	1.322	1.363 [0.541, 2.185]
Any AI vs. D, mean difference	0.898	0.911	0.962 [0.268, 1.655]
Arm × tendency interaction, *F*	7.248	7.264	6.171 [4.335, 7.846]

Differences are in transfer-rubric points (0–24). Inverse-probability weights come from a logistic model of post-test completion on arm and all four baseline measures; that model does not fit better than an intercept-only model; likelihood-ratio *p* = 0.859, and the resulting weights range from 1.08 to 1.26. Multiple imputation uses 40 imputations by chained equations with each arm and all baseline measures in the imputation model; brackets present 95% confidence intervals pooled using Rubin’s rules, and the interval for *F* provides the 2.5th and 97.5th percentiles across imputations. Complete-case and imputed point estimates agree to within 0.08 points on every contrast.

**Table 3:** Pre-specified contrasts on transfer performance (Site A completers, *n* = 639).

Contrast	Comparison	Cohen’s *d* [95% CI]	*t*	Holm *p*	Status
*Panel A. The three contrasts specified in the analysis plan*					
C1	A (AI drafts) vs. B (AI evals)	0.35 [0.13, 0.57]	3.16	0.004	planned
C2	C (AI revises) vs. B (AI evals)	0.36 [0.14, 0.58]	3.21	0.004	planned
C3	Any AI (A, B, C) vs. D (no AI)	0.23 [0.05, 0.41]	2.51	0.012	planned
*Panel B. All six pairwise contrasts, Holm-adjusted across the six*					
	A (AI drafts) vs. B (AI evals)	0.35 [0.13, 0.57]	3.16	0.009	planned
	A (AI drafts) vs. C (AI revises)	0.02 [−0.20, 0.24]	0.21	1.000	added
	A (AI drafts) vs. D (no AI)	0.35 [0.13, 0.57]	3.12	0.009	added
	B (AI evals) vs. C (AI revises)	−0.36 [−0.58, −0.14]	−3.21	0.009	planned
	B (AI evals) vs. D (no AI)	−0.004 [−0.22, 0.22]	−0.04	1.000	added
	C (AI revises) vs. D (no AI)	0.36 [0.13, 0.58]	3.17	0.009	added
*Panel C. Equivalence tests (TOST, bounds ± 0.30 d)*					
	A vs. C	*d* = 0.02, difference = 0.09 points		0.007	added
	B vs. D	*d* = −0.004 [−0.22, 0.22]		0.004	planned

Omnibus test: *F*(3, 634) = 8.18, *p* < 0.001, $\eta_{p}^{2}$ = 0.037, baseline writing ability as covariate. In the Status column, “planned” marks a contrast fixed in the analysis plan and “added” one introduced after the plan was fixed. Panel A reports the three planned contrasts, Holm-adjusted within that family. Panel B reports every pairwise contrast, since H1a as stated requires each AI arm to exceed the control individually; Holm adjustment there is across all six, which is why the two *p*-values common to both panels differ. Panel C reports TOST *p* values, both supporting equivalence. H1a is supported in two of three components: arms A and C each exceeded the control, arm B did not and is statistically equivalent to it, and the combined-AI contrast (*d* = 0.23) falls below the pre-set smallest effect size of interest of 0.30 because it averages over an arm that conferred no benefit.

**Table 4:** Peer-review quality by arm (Site A completers, *n* = 639).

	Arm A	Arm B	Arm C	Arm D	*F*(3, 634)	Holm *p*	$\eta_{p}^{2}$
Problem identification	2.68 (0.78)	2.50 (0.75)	2.48 (0.77)	2.52 (0.86)	2.37	0.070	0.011
Problem explanation	2.77 (0.85)	2.43 (0.78)	2.47 (0.85)	2.48 (0.83)	5.96	0.002	0.027
Constructive suggestion	2.74 (0.85)	2.52 (0.82)	2.51 (0.77)	2.49 (0.84)	3.58	0.027	0.017
*H1b contrasts: arm A against each comparison arm, Holm-adjusted within the pre-specified family*							
Problem identification	A vs. B: *d* = 0.24 [0.02, 0.46], *p* = 0.139			A vs. C: *d* = 0.26 [0.04, 0.48], *p* = 0.125			
Problem explanation	A vs. B: *d* = 0.41 [0.19, 0.63], *p* = 0.003			A vs. C: *d* = 0.35 [0.13, 0.57], *p* = 0.017			
Constructive suggestion	A vs. B: *d* = 0.26 [0.04, 0.48], *p* = 0.125			A vs. C: *d* = 0.28 [0.06, 0.50], *p* = 0.089			
*Arm B versus no-AI control (D): equivalence against ±0.30 d*							
Problem identification	*d* = −0.03 [−0.24, 0.19]				TOST *p* = 0.007 (equivalent)		
Problem explanation	*d* = −0.05 [−0.27, 0.17]				TOST *p* = 0.014 (equivalent)		
Constructive suggestion	*d* = 0.04 [−0.18, 0.26]				TOST *p* = 0.011 (equivalent)		

Cell entries in the upper panel are *M* (*SD*) on 0–5 scales. Multivariate test: Wilks’ Λ = 0.967, *F*(9, 1538.3) = 2.39, *p* = 0.011. The A versus C contrasts are Holm-adjusted together with the A versus B and B versus D contrasts, with nine tests in the pre-specified family. All six A-versus-comparison contrasts run in the predicted direction; only the problem explanation survives correction against both comparison arms.

**Table 5:** Simple slopes of dependent offloading tendency on transfer performance by arm.

Arm	*n*	*b* [95% CI]	*SE*	*p*
A (AI drafts)	158	−1.41 [−1.97, −0.85]	0.28	<0.001
B (AI evaluates)	162	−0.07 [−0.62, 0.48]	0.28	0.806
C (AI revises)	160	0.23 [−0.27, 0.72]	0.25	0.367
D (no AI)	159	−0.10 [−0.66, 0.45]	0.28	0.719

Interaction test: *F*(3, 629) = 7.25, *p* < 0.001, $\eta_{p}^{2}$ = 0.033. Models control autonomous tendency and baseline ability. Johnson–Neyman: the arm A advantage over arm D ceases to be distinguishable from zero above a dependent tendency of 3.45 (sample *M* = 2.93, *SD* = 1.08).

**Table 6:** (**a**) Error detection by planted-error type (Site A completers, *n* = 639). (**b**) All pairwise comparisons on the catch rate (Holm-adjusted).

(**a**)						
**Error Type**	**Overall Rate**	**Arm A**	**Arm B**	**Arm C**	**Arm D**	***d*** **(A vs. D) [95% CI]**
Fabricated citation	0.529	2.49	2.05	2.01	1.92	0.51 [0.29, 0.74]
Logical fallacy	0.533	2.36	2.04	2.03	2.10	0.23 [0.00, 0.45]
Evidence–conclusion mismatch	0.555	2.50	2.21	2.06	2.11	0.34 [0.12, 0.57]
All 12 errors (catch rate)	0.539	0.612	0.525	0.508	0.511	0.48 [0.26, 0.70]
(**b**)						
**Comparison**	**Cohen’s** ***d*** **[95% CI]**		**Holm** ***p***		**TOST** ***p*** **(±0.30** ***d*****)**	
A vs. B	0.42 [0.20, 0.65]		<0.001		—	
A vs. C	0.51 [0.28, 0.73]		<0.001		—	
A vs. D	0.48 [0.26, 0.70]		<0.001		—	
B vs. C	0.09 [−0.13, 0.30]		1.000		0.027 (equivalent)	
B vs. D	0.07 [−0.15, 0.29]		1.000		—	
C vs. D	−0.02 [−0.24, 0.20]		1.000		0.006 (equivalent)	

(**a**) Arm means are counts out of four per type. All arm effects are reliable after Benjamini–Hochberg correction (*p* < 0.001, 0.021, and 0.002, respectively); models control the baseline ability and false-positive rate. (**b**) Arm A exceeds every other arm; arm C, in which the model performed the revision, is statistically equivalent to the no-AI control on detection despite matching arm A on transfer.

**Table 7:** Individual-difference predictors of error-catch rate (Site A completers, *n* = 639).

Predictor	*b*	*SE*	95% CI	*p*
Dependent offloading tendency	−0.042	0.008	[−0.057, −0.027]	<0.001
Autonomous offloading tendency	−0.004	0.007	[−0.018, 0.011]	0.610
Metacognitive monitoring	0.007	0.011	[−0.016, 0.029]	0.571

Model controls baseline writing ability and false-positive rate. Benjamini–Hochberg correction applied within the detection family.

## Data Availability

The analysis plan and the analysis code are provided as Supplementary Material and require no request. The instrument materials—the prompt templates, the rubric, the four report topics, and the planted-error manuscript with its scoring key—are available from the corresponding author on reasonable request rather than posted openly, because this research programme is ongoing and continues to use them. The planted-error instrument in particular would be compromised by publication, since it functions only while the errors it contains are unknown to prospective participants, and the same consideration applies to the topics and rubric, which remain in use in this research programme. Participant-level data are not publicly available: the consent obtained from participants and the agreements with the participating institutions do not permit onward distribution of individual-level records, including in de-identified form. De-identified datasets are available from the corresponding author on reasonable request, subject to those conditions and to approval by the participating institutions.

## Supplementary Materials

The following supporting information can be downloaded at https://www.mdpi.com/article/10.3390/bs16091671/s1, the analysis plan fixed before data collection; the model used in the AI arms together with its version, access mode and configuration; the full analysis scripts, including those for the sensitivity and robustness analyses reported here, together with the estimates they produce and the console log of a complete run; and the coding scheme in Appendix A in machine-readable form.

## Appendix A. Prompt Coding Scheme for the Re-Outsourcing Ratio

Every prompt a participant sent to the model was assigned to exactly one of the four categories below. The unit of coding is the prompt, not the session; the re-outsourcing ratio reported in the main text is the proportion of a participant’s prompts falling into category R.

Three conventions settled the cases that generated disagreement at the double-coding stage. First, a request framed as a question but answerable only by a verdict is coded R, not L: *do you think this works?* asks for the judgement, however it is phrased. Second, a prompt that asks both for a verdict and for its rationale is coded R, on the ground that the verdict has already been outsourced, whether or not the reasoning accompanies it. Third, a prompt asking the model to check a specific, participant-nominated property is coded L when the participant has stated the criterion (*does paragraph 2 provide evidence for the claim about sample size?*) and R when they have not (*check whether the evidence is adequate*).

**Table A1:** Categories, decision rules, and examples. Examples are translated from the original Chinese and lightly edited for length.

Category	Decision Rule	Examples
**R. Re-delegating judgement**	The prompt asks the model to supply an evaluative verdict, or to enact a change, in place of the participant making or specifying it. The test is whether a compliant response would leave the participant with a judgement they did not have to form.	*What is wrong with this paragraph? Evaluate this text and tell me what errors it has. Just fix it for me. Is this argument good enough? Rewrite the conclusion so it works.*
**L. Learning-oriented**	The prompt asks for explanation, justification, illustration, or background that the participant would then apply themselves. A compliant response informs a judgement the participant still has to make.	*Why would a reviewer consider this evidence weak? Give me an example of a well-formed causal claim. Explain the difference between these two citation formats.*
**T. Task management**	The prompt concerns logistics, format, word count, or the mechanics of the platform, with no evaluative or substantive content.	*How long should Section 3 be? Put the references in APA style. Can I submit a draft in two files?*
**U. Uncodable**	Fragmentary, empty, off-task, or ambiguous between R and L after the decision rules are applied. Retained in the denominator.	*ok continue* off-topic content

Category R is interpretable as re-delegation only where the participant had been assigned evaluative work that could be forwarded. That condition holds in arm A. In arm B, the participant had no assigned evaluative task, and in arm C, adjudication was completed inside a structured platform form with no channel to the model, so the ratio is structurally zero in both; in arm D, it is undefined. This asymmetry is a property of the workflows rather than of the participants, and the main text treats it as such.

## Appendix B. Supporting Detail for the Design, Measures, and Robustness Analyses

### Appendix B.1. Sample Size

Power was determined by Monte Carlo simulation of the planned ANCOVA, 2000 replications per cell, α = 0.05, with the arm × tendency interaction as the primary test. For an interaction of Cohen’s *f* = 0.20, power was 0.42 at 55 per arm, 0.64 at 90 and 0.82 at 130; at 130 per arm, power was 0.54 for *f* = 0.15 and 0.95 for *f* = 0.25. Detection was not the binding constraint, reaching 0.96 at 25 per arm because each participant contributes twelve binary judgements, and the reduced replication at Site B was powered at 0.85 for *d* = 0.8 with 35 per arm. The simulation modelled observed effects as attenuated by the reliability of the measures rather than assuming error-free measurement; on the same basis, letting rater ICC fall from 0.90 to 0.55 reduces power for a *d* = 0.50 effect from 0.68 to 0.49, which is why the rating protocol treated its ICC threshold as a gate.

### Appendix B.2. Allocation

Strata were baseline writing tertile crossed with faculty classification, six per site. Within each stratum, the platform randomly permuted the students and divided them among the arms, so in terms of block randomisation, the block spans the whole stratum rather than being a fixed small number. Where a stratum did not divide exactly, the remainder fell to arms determined by the permutation, leaving at most one participant of difference within that stratum; across strata, these were offset, and the realised arm totals were exactly equal (Table A2).

**Table A2:** Allocation by stratum and arm.

Stratum	Arm A	Arm B	Arm C	Arm D	Total
*Site A*					
Tertile 1, other	26	26	26	26	104
Tertile 1, social science	36	36	36	36	144
Tertile 2, other	19	20	19	20	78
Tertile 2, social science	43	42	43	42	170
Tertile 3, other	27	27	27	27	108
Tertile 3, social science	35	35	35	35	140
*Total*	186	186	186	186	744
*Site B*					
Tertile 1, other	5	5	—	6	16
Tertile 1, social science	9	9	—	8	26
Tertile 2, other	5	6	—	5	16
Tertile 2, social science	9	9	—	9	27
Tertile 3, other	5	4	—	5	14
Tertile 3, social science	9	9	—	9	27
*Total*	42	42	—	42	126

### Appendix B.3. Sample Composition and Platform Use

By faculty classification, the stratifying variable, 454 of 744 participants at Site A (61.0%) and 80 of 126 at Site B (63.5%), fell in the social-science category and the remainder in the other. Baseline frequency of generative AI use was nearly identical at the two sites on a five-point scale, *M* = 2.96, *SD* = 1.08 at Site A and *M* = 2.96, *SD* = 1.15 at Site B, and did not differ across arms.

Prompt volumes over the four rounds were comparable across the AI arms: arm A, *M* = 19.68, range 10–30; arm B, *M* = 20.12, range 9–33; arm C, *M* = 19.99, range 11–32. No participant in arm D produced any interaction, and no arm D participant was identified as having used AI by either logs or self-report.

### Appendix B.4. Measurement Reliability

Inter-rater reliability, two-way random effects, absolute agreement, and three raters: baseline writing, ICC(2,*k*) = 0.787, 95% CI [0.759, 0.812] at Site A and 0.846 [0.792, 0.887] at Site B; transfer performance, 0.831 [0.806, 0.852] and 0.826 [0.760, 0.876]; peer-review dimensions at Site A, 0.824 for problem identification, 0.821 for explanation and 0.819 for suggestion. Single-rater coefficients, ICC(2,1), range from 0.551 to 0.646; all analyses use the three-rater mean. Rater main effects were not reliable for any outcome: largest *F* = 1.88, *p* = 0.153.

Offloading subscales: dependent tendency, α = 0.807 and ω = 0.807 at Site A, and α = 0.792 and ω = 0.793 at Site B; autonomous tendency, α = 0.806 and ω = 0.806 at Site A, and α = 0.827 and ω = 0.829 at Site B. No item improved either scale by its deletion.

Detection instrument: item pass rates ranged from 0.32 to 0.75 and the three type-level rates from 0.516 to 0.555, within the band the piloting targeted. KR-20 was 0.619 [0.574, 0.662] at Site A and 0.523 [0.380, 0.646] at Site B. Within the four-item type subsets the coefficient falls to between 0.20 and 0.38; the mean inter-item correlation within types, 0.11 to 0.14 at Site A, is of the same order as that across the full twelve, 0.12, so the reduction reflects test length rather than heterogeneity of content.

### Appendix B.5. Missing Outcome Data

A logistic model of post-test completion on arm and all four baseline measures fitted no better than an intercept-only model, likelihood-ratio *p* = 0.859, with no predictor approaching reliability; the implied inverse-probability weights range from 1.08 to 1.26. Under those weights, the arm contrasts shift in the third decimal place—the B versus A coefficient moves from −1.395 to −1.407—and the arm × tendency interaction from *F* = 7.248 to *F* = 7.264. Multiple imputation used 40 imputations by chained equations with arm and all baseline measures in the imputation model, pooled by Rubin’s rules; pooled differences and intervals appear in Table 2, and the interaction remained reliable across imputations.

### Appendix B.6. The Detection Outcome as a Binomial Count

A binomial generalised linear model with a logit link, twelve trials per participant and the same covariates reproduces the arm effect, likelihood-ratio $\chi^{2}$(3) = 57.70, *p* < 0.001. Relative to arm A the odds of catching a given error were 0.70 [0.61, 0.80] in arm B, 0.65 [0.57, 0.74] in arm C and 0.65 [0.57, 0.74] in arm D. Entering error type as a within-participant factor in a GEE model with an exchangeable working correlation shows no arm × type interaction, Wald $\chi^{2}$(6) = 5.69, *p* = 0.459, while the arm effect holds.

### Appendix B.7. Robustness Checks Specified in the Plan

*Rater sensitivity.* The arm × tendency interaction does not depend on the rating aggregation. Re-estimated on each rater’s scores singly it remains reliable—*F*(3, 629) = 4.68, *p* = 0.003 for the first, 6.10, *p* < 0.001 for the second, and 4.47, *p* = 0.004 for the third—as it does on two-rater means (*F* = 6.97 and 5.93, both *p* < 0.001), against *F* = 7.25 on the three-rater mean used throughout.

*Model specification.* The plan’s model included faculty classification alongside baseline ability as a fixed covariate. Adding it changes the arm effect from *F*(3, 634) = 8.18 to 8.15 and the arm × tendency interaction from *F*(3, 629) = 7.25 to 7.22; the models reported in the text use the more parsimonious specification. With two categories, faculty classification adjusts for the stratifying variable and does not represent class- or instructor-level dependence.

*Link function for the proportion and count outcomes.* The plan specified a quasi-binomial model for the re-outsourcing ratio and a binomial model for the individual-difference analysis of catch rate; the text reports both on the linear scale, where the coefficients are directly interpretable in the units of the measures. Under the planned link functions, the conclusions are identical: dependent tendency predicts re-outsourcing at *b* = 0.360 in log odds, *p* < 0.001, and predicts catch rate at *b* = −0.173, odds ratio 0.841, *p* < 0.001, with autonomous tendency (*p* = 0.458) and metacognitive monitoring (*p* = 0.374) again predicting neither.

*Scale structure.* Both offloading subscales are single-factor in these samples. For dependent tendency, the first eigenvalue is 2.52 against 0.51 for the second, accounting for 63.1% of item variance with loadings of 0.79 to 0.80; for autonomous tendency, the corresponding values are 2.54 and 0.52, 63.6%, and loadings of 0.79 to 0.81.

### Appendix B.8. Pooled Two-Site Analysis

With site as a fixed-effect covariate, the arm × tendency interaction was reliable (*F*(2, 579) = 7.00, *p* = 0.001) alongside the main effect of the arm (*F*(2, 579) = 11.27, *p* < 0.001). The site contributed nothing once baseline ability was in the model (*F*(1, 579) = 0.27, *p* = 0.603), and the arm × site interaction was negligible (*F*(2, 581) = 0.13, *p* = 0.880).

## References

[B1-behavsci-16-01671] Alghamdi L. H., Alghizzi T. M. (2026). Metacognitive filtering and cognitive offloading in AI-assisted L2 writing: A PRISMA guided process-tracing synthesis. Behavioral Sciences.

[B2-behavsci-16-01671] Bearman M., Tai J., Dawson P., Boud D., Ajjawi R. (2024). Developing evaluative judgement for a time of generative artificial intelligence. Assessment & Evaluation in Higher Education.

[B3-behavsci-16-01671] Bjork E. L., Bjork R. A., Gernsbacher M. A., Pew R. W., Hough L. M., Pomerantz J. R. (2011). Making things hard on yourself, but in a good way: Creating desirable difficulties to enhance learning. Psychology and the real world: Essays illustrating fundamental contributions to society.

[B4-behavsci-16-01671] Bjork R. A., Dunlosky J., Kornell N. (2013). Self-regulated learning: Beliefs, techniques, and illusions. Annual Review of Psychology.

[B5-behavsci-16-01671] Buçinca Z., Malaya M. B., Gajos K. Z. (2021). To trust or to think: Cognitive forcing functions can reduce overreliance on AI in AI-assisted decision-making. Proceedings of the ACM on Human-Computer Interaction.

[B6-behavsci-16-01671] Carless D., Boud D. (2018). The development of student feedback literacy: Enabling uptake of feedback. Assessment & Evaluation in Higher Education.

[B7-behavsci-16-01671] Fan W., Cheng L., Wang Y., Zhao Q., Li Y. (2026). In-class AI use and attitudes among university students: The different mediating roles of cognitive relief and cognitive offloading. Behavioral Sciences.

[B8-behavsci-16-01671] Fan Y., Tang L., Le H., Shen K., Tan S., Zhao Y., Shen Y., Li X., Gašević D. (2025). Beware of metacognitive laziness: Effects of generative artificial intelligence on learning motivation, processes, and performance. British Journal of Educational Technology.

[B9-behavsci-16-01671] Fleming S. M., Lau H. C. (2014). How to measure metacognition. Frontiers in Human Neuroscience.

[B10-behavsci-16-01671] Grinschgl S., Papenmeier F., Meyerhoff H. S. (2023). Mutual interplay between cognitive offloading and secondary task performance. Psychonomic Bulletin & Review.

[B11-behavsci-16-01671] Jakesch M., Hancock J. T., Naaman M. (2023). Human heuristics for AI-generated language are flawed. Proceedings of the National Academy of Sciences.

[B12-behavsci-16-01671] Kaplar M., Luzanin Z., Vucic M., Ivanovic L., Kaplar S. (2026). Engineering students’ critical engagement with ChatGPT: Effects of output accuracy on answers and confidence in a real-world probability context. IEEE Transactions on Education.

[B13-behavsci-16-01671] Kelemen W. L., Frost P. J., Weaver C. A. (2000). Individual differences in metacognition: Evidence against a general metacognitive ability. Memory & Cognition.

[B14-behavsci-16-01671] Kirschner P. A., Sweller J., Clark R. E. (2006). Why minimal guidance during instruction does not work: An analysis of the failure of constructivist, discovery, problem-based, experiential, and inquiry-based teaching. Educational Psychologist.

[B15-behavsci-16-01671] Koriat A. (1997). Monitoring one’s own knowledge during study: A cue-utilization approach to judgments of learning. Journal of Experimental Psychology: General.

[B16-behavsci-16-01671] Koriat A., Bjork R. A. (2005). Illusions of competence in monitoring one’s knowledge during study. Journal of Experimental Psychology: Learning, Memory, and Cognition.

[B17-behavsci-16-01671] Kruger J., Dunning D. (1999). Unskilled and unaware of it: How difficulties in recognizing one’s own incompetence lead to inflated self-assessments. Journal of Personality and Social Psychology.

[B18-behavsci-16-01671] Lai C., Pan M., Guo K., Cui Y. (2026). What task to offload to GenAI in the writing feedback process? Effects of task-offloading approaches on EFL learners’ writing skill development. Computers & Education.

[B19-behavsci-16-01671] Liu S., Kent C., Briscoe J. (2025). Monitoring-based rewards enhance both learning performance and metacognitive monitoring accuracy. Behavioral Sciences.

[B20-behavsci-16-01671] Messeri L., Crockett M. J. (2024). Artificial intelligence and illusions of understanding in scientific research. Nature.

[B21-behavsci-16-01671] Molloy E., Boud D., Henderson M. (2020). Developing a learning-centred framework for feedback literacy. Assessment & Evaluation in Higher Education.

[B22-behavsci-16-01671] Ng D. T. K., Leung J. K. L., Chu S. K. W., Qiao M. S. (2021). Conceptualizing AI literacy: An exploratory review. Computers and Education: Artificial Intelligence.

[B23-behavsci-16-01671] Parasuraman R., Manzey D. H. (2010). Complacency and bias in human use of automation: An attentional integration. Human Factors.

[B24-behavsci-16-01671] Risko E. F., Gilbert S. J. (2016). Cognitive offloading. Trends in Cognitive Sciences.

[B25-behavsci-16-01671] Roediger H. L., Karpicke J. D. (2006). Test-enhanced learning: Taking memory tests improves long-term retention. Psychological Science.

[B26-behavsci-16-01671] Schraw G. (2009). A conceptual analysis of five measures of metacognitive monitoring. Metacognition and Learning.

[B27-behavsci-16-01671] Silva J. B., Albuquerque P. B., Oliveira I. B., Rodrigues P. F. S. (2026). Between alarms and scheduling: The effect of cognitive offloading on prospective and retrospective memory. Behavioral Sciences.

[B28-behavsci-16-01671] Skitka L. J., Mosier K. L., Burdick M. (1999). Does automation bias decision-making?. International Journal of Human-Computer Studies.

[B29-behavsci-16-01671] Skulmowski A. (2023). The cognitive architecture of digital externalization. Educational Psychology Review.

[B30-behavsci-16-01671] Slamecka N. J., Graf P. (1978). The generation effect: Delineation of a phenomenon. Journal of Experimental Psychology: Human Learning and Memory.

[B31-behavsci-16-01671] Sparrow B., Liu J., Wegner D. M. (2011). Google effects on memory: Cognitive consequences of having information at our fingertips. Science.

[B32-behavsci-16-01671] Sweller J. (1988). Cognitive load during problem solving: Effects on learning. Cognitive Science.

[B33-behavsci-16-01671] Tai J., Ajjawi R., Boud D., Dawson P., Panadero E. (2018). Developing evaluative judgement: Enabling students to make decisions about the quality of work. Higher Education.

[B34-behavsci-16-01671] Tang M., Lu T., You X. (2026). From initial to situational automation trust: The interplay of personality, interpersonal trust, and trust calibration in young males. Behavioral Sciences.

[B35-behavsci-16-01671] Walters W. H., Wilder E. I. (2023). Fabrication and errors in the bibliographic citations generated by ChatGPT. Scientific Reports.

[B36-behavsci-16-01671] Wang G., Wang W., Yang D., Ren J. (2026). Generative AI, cognitive offloading, and learner agency in higher education: A scoping review. Behavioral Sciences.

[B37-behavsci-16-01671] Wang Y., Mi X., Tang W., Tang Y., Gao H. (2026). How generative AI use styles shape academic engagement: The roles of academic impostor syndrome and AI policy clarity. Behavioral Sciences.

[B38-behavsci-16-01671] Yildirim-Erbasli S. N., Dibek M. I., Thomas M. L., Lesoway N. (2026). When do undergraduate students prefer AI? Insights into AI scoring and feedback. Behavioral Sciences.

[B39-behavsci-16-01671] Zhang W., Zhang Z., Chang P., Zhu Q., Wang D. (2026). Psychological consequences of AI-assisted training and the buffering role of mindfulness. Acta Psychologica.

[B40-behavsci-16-01671] Zhu Q., Li X., Dong Y., Chang P., Fan M. (2026). Not all cognitive offloading is equal: Distinguishing dependent and autonomous offloading to generative AI. Frontiers in Psychology.

[B41-behavsci-16-01671] Zimmerman B. J. (2002). Becoming a self-regulated learner: An overview. Theory into Practice.

