# Supplementary material for: Telling Students to Evaluate Does Not Make It Happen: Task Stage, Offloading Tendency, and Error Detection in AI-Assisted Student Writing
Source: Behav Sci (Basel). 2026 Sep 17;16(9):1671. doi: 10.3390/bs16091671 (PMC13603190; doi:10.3390/bs16091671)
Supplement: Supplementary file 1 [file behavsci-16-01671-s001.zip › supplement-analysis-plan.pdf]

Analysis Plan

Supplementary Material accompanying the manuscript *Telling Students to Evaluate Does Not Make It Happen: Task Stage, Offloading Tendency, and Error Detection in AI-Assisted Student Writing* (submitted to *Behavioral Sciences*), stating how the study's analysis is organised. The plan is fixed within the research team before data collection and carries no third-party timestamp; the analyses are pre-specified rather than preregistered.

1. Design and sample

The experiment is a four-arm, between-participants randomised design over a fixed three-stage writing cycle (draft, evaluate, revise). The AI drafts in arm A, leaving evaluation and revision to the student; evaluates in arm B; and revises in arm C, the student adjudicating each change. Arm D works without AI.

Site A supplies the primary sample and the full four-arm protocol; Site B a reduced three-arm conceptual replication (arms A, B and D, without the peer-review task). All primary tests are within site.

Randomisation is at the individual level, separately at each site, within strata defined by baseline writing tertile crossed with faculty classification (six per site); the sequence is generated by the course platform and concealed until baseline measurement is complete.

Sample size is fixed in advance by Monte Carlo simulation (2,000 replications per cell, $\alpha = 0.05$) of the primary test, the three-degree-of-freedom arm × tendency interaction: 130 completers per arm gives power of 0.82 for Cohen's $f = 0.20$. Site A randomises 744 students (186 per arm), Site B 126 (42 per arm), and participants are analysed as randomised, with no reassignment by compliance.

2. Outcomes and measurement

Transfer performance (primary outcome): an unaided report on a new topic, rated on a six-dimension rubric, 0–24. **Peer-review quality**: a common sample manuscript evaluated on problem identification, problem explanation and constructive suggestion, each scored 0–5 (Site A only). **Error detection**: catch rate, the proportion of 12 planted errors identified (three types, four each), scored against a fixed key by assistants blind to condition; false positives are recorded likewise and enter the models as a covariate. Internal consistency is assessed by KR-20; confirmatory analyses use the twelve-item total, with results by error type exploratory.

Rubric-scored outcomes are rated blind to condition and phase, rating beginning once $ICC(2,k) \geq 0.75$ is reached on a calibration set. Each script receives three ratings, and a fourth whenever two raters differ by two points or more; analyses use the three-rater mean. Reliability is reported per outcome and site as $ICC(2,k)$, two-way random effects, absolute agreement, with rater main effects tested.

Interaction logs yield the re-outsourcing ratio: the proportion of a participant's prompts — the prompt is the coding unit — classified as re-delegating judgement rather than seeking explanation. Two coders independently code a random 20%, agreement is assessed by Cohen's κ against the protocol threshold, disagreements resolved by discussion, and one coder codes the remainder under that scheme (Appendix A). The ratio is informative in arm A, structurally zero in arms B and C, undefined in D.

Self-report: dependent and autonomous offloading tendency (four-item subscales) and metacognitive monitoring, each summarised by Cronbach's α and McDonald's ω; the post-measures immediate satisfaction and self-rated quality are single items. **Baseline writing ability**, an unaided 800-word note rated under the same blinding, is both stratifying variable and covariate.

3. Confirmatory tests

H1a. ANCOVA of transfer on arm with baseline ability as covariate, followed by three pre-specified contrasts: A versus B, C versus B, and the mean of the AI arms versus D.

H1b. MANOVA (Wilks' Λ) across the three peer-review dimensions, with univariate follow-ups, the pre-specified contrasts A versus B and A versus C on each dimension, and arm B against the no-AI control.

H2. The arm × centred dependent tendency interaction, with autonomous tendency also in the model since the two are distinct; tendency is never dichotomised. A reliable interaction is followed by within-arm simple slopes and a Johnson–Neyman region of significance. Within arm A, the re-outsourcing ratio is regressed on dependent tendency and correlated with transfer, and the indirect path through it is bootstrapped.

H3. (a) The arm effect on catch rate with false positives controlled; (b) catch rate against immediate satisfaction and self-rated quality, as raw correlations and adjusted for arm and baseline ability, with self-rated quality against transfer as the reference association; (c) catch rate regressed on dependent tendency, autonomous tendency and metacognitive monitoring, with baseline ability controlled.

Balance is checked by a one-way test of arm on each baseline measure, and attrition before outcomes are examined: completion is tested against arm, and completers and non-completers compared on every baseline measure. Compliance in arm D is verified against platform logs and self-report.

4. Multiple-comparison families

Correction is applied within families: Holm for the transfer-contrast and peer-review families, Benjamini–Hochberg for the detection family. The single pre-specified interaction test is not corrected, a decision fixed in advance.

5. Effect sizes and the smallest effect size of interest

Effect sizes are point estimates with 95% confidence intervals — partial η^2 for omnibus tests, Cohen's d for contrasts — and p values are not the sole basis for any conclusion. A smallest effect size of interest of $d = 0.30$ is set in advance; effects below it are reported on both scales even when statistically reliable.

6. Equivalence testing

Where a null carries interpretive weight, equivalence is tested by two one-sided tests against bounds of ±0.30 d, or ±0.10 for correlations: arm B against the no-AI control on transfer and on each peer-review dimension, arm C against the no-AI control and against arm B on detection, and catch rate against immediate satisfaction.

7. Missing outcome data

Outcomes are measured only at the post-test, so primary analyses are complete-case by randomised assignment. Completion is modelled logistically on arm and all four baseline measures; the primary models are re-estimated with inverse-probability weights from it, and after multiple imputation by chained equations (40 imputations, arm and all baseline measures in the imputation model, pooled by Rubin's rules).

8. Robustness checks

- Rater sensitivity: the interaction re-estimated on single-rater scores and on two-rater means.
- Model specification: the interaction re-estimated without covariates and with faculty classification added as a fixed covariate; plus the arm × baseline ability interaction, testing homogeneity of regression slopes.
- Link functions: quasi-binomial for the re-outsourcing ratio and binomial for the catch-rate individual-difference model, alongside the linear-scale versions; a binomial GLM with logit link, twelve trials per participant, for the arm effect on detection; a GEE with exchangeable working correlation entering error type as a within-participant factor.
- Scale structure: single-factor checks on both offloading subscales and the correlation between them.
- Conceptual replication: the stage main effect and interaction re-estimated at Site B.
- Pooled two-site analysis of the three common arms with site as a fixed-effect covariate.

Analyses added after the data were seen — the six pairwise transfer contrasts under Holm correction, the equivalence of arms A and C on transfer, the detection breakdown by error type, and the pooled two-site analysis — are exploratory.

9. Software

Analyses use Python (`statsmodels`, `scipy`); the bootstrap uses 5,000 resamples under a fixed seed. The analysis scripts accompany this plan, so every estimate traces to its specification and code.
