# Supplementary material for: Telling Students to Evaluate Does Not Make It Happen: Task Stage, Offloading Tendency, and Error Detection in AI-Assisted Student Writing
Source: Behav Sci (Basel). 2026 Sep 17;16(9):1671. doi: 10.3390/bs16091671 (PMC13603190; doi:10.3390/bs16091671)
Supplement: Supplementary file 1 [file behavsci-16-01671-s001.zip › supplementary-materials.pdf]

Supplementary Materials

Manuscript ID: behavsci-4543232 **Title:** Telling Students to Evaluate Does Not Make It Happen: Task Stage, Offloading Tendency, and Error Detection in AI-Assisted Student Writing

---

S1. The model used in the AI arms and its configuration

All three AI arms used the same model, the same version, the same configuration and the same prompt-template pool. The arms differed only in which stage of the writing cycle the model performed and in what the student was required to do with its output. The model is therefore held constant across arms A, B and C by design, and cannot account for the differences among them.

The model was Qwen 3.0, supplied by Alibaba Cloud and reached through the study platform's server-side interface rather than through a consumer chat application. Decoding used the provider's default settings; no parameter was altered for this study. Web search and retrieval over external sources were disabled and no external tools were available. The provider's persistent-memory feature was enabled. This is not the same as carrying the full transcript of earlier rounds into the context window: the system stored salient information distilled from a student's previous interactions and made it available to the model in later rounds. Each student's memory store was specific to that student. The feature was enabled identically in arms A, B and C, and it reproduces the way students ordinarily work with such a system across a term rather than starting afresh at each assignment. Participants could not select or change the model, alter its settings, or view or modify the system instruction.

The interface language was Chinese, as were all tasks, rubrics, prompts and student texts.

S2. System instruction and prompt templates

Prompting was templated rather than free-form at the point where the model performed its assigned stage: the platform composed the request from a fixed template and the student's own text. Where the workflow gave the student free interaction with the model — in arm A, after the draft was returned — prompts were the student's own, and it is those prompts that the re-outsourcing measure codes (see S5).

The templates themselves are not reproduced here. They are part of the instrument set for a research programme that is still running, and are available from the corresponding author on reasonable request.

S3. Analysis plan

The plan sets out the design and sample, the outcomes and how each is scored, the model for each hypothesis, the correction families, the smallest effect size of interest, where equivalence is tested and against what bounds, the treatment of missing outcome data, and the robustness checks. Analyses added after the data were seen are identified as such in the manuscript wherever they are reported.

The plan is provided as a separate file, `supplement-analysis-plan.md`.

S4. Task materials

The six-dimension rubric, the four report topics, the transfer task, the sample manuscript used for the peer-review task, and the planted-error instrument with its scoring key are available from the corresponding author on reasonable request. They are not posted openly: the planted-error instrument functions only while the errors it contains are unknown to prospective participants, and the topics and rubric remain in use in this research programme.

S5. Prompt coding scheme

Reproduced as Appendix A of the manuscript, with the four categories, their decision rules, worked examples, and the three conventions that settled borderline cases at the double-coding stage. A machine-readable version accompanies these materials as `prompt-coding-scheme.tsv` and `prompt-coding-conventions.txt`.

S6. Analysis scripts and outputs

`analysis_main.py` runs all primary, secondary and robustness analyses in the order reported. `analysis_revision.py` adds the full set of pairwise contrasts, the missing-data sensitivity analyses by inverse-probability weighting and multiple imputation, the binomial GLM and the GEE model for the detection outcome, the pooled two-site analysis with site as a fixed effect, and the simple-slope grid. `reliability_analysis.py` produces the inter-rater ICCs, the scale alpha and omega coefficients, and the KR-20 and item statistics for the detection instrument. `make_figures.py` produces all six figures.

The directories `results/`, `results_revision/` and `results_reliability/` hold every estimate reported in the manuscript as CSV and JSON, together with the full console log of one complete run of each script.

Analyses used Python 3.12. Scripts require Python 3.9 or later with `pandas`, `numpy`, `scipy`, `statsmodels`, `matplotlib` and `openpyxl`. The exact historical analysis-package versions were not recorded; the official package websites are https://www.statsmodels.org/ and https://scipy.org/ (both accessed on 17 September 2026). The bootstrap for the indirect effect uses a fixed seed (20260814) and reproduces exactly.

Participant-level data are not included: the consent obtained from participants and the agreements with the participating institutions do not permit onward distribution of individual-level records, including in de-identified form. See the Data Availability Statement.
